# Investigating Functional Roles for Positive Feedback and Cellular Heterogeneity in the Type I Interferon Response to Viral Infection

**DOI:** 10.3390/v10100517

**Published:** 2018-09-21

**Authors:** Sivan Leviyang, Igor Griva

**Affiliations:** 1 Department of Mathematics and Statistics, Georgetown University, Washington, DC 20057, USA; 2 Department of Mathematical Sciences, George Mason University, Fairfax, VA 22030, USA; igriva@gmu.edu

**Keywords:** interferon, virus, heterogeneity, innate immunity

## Abstract

Secretion of type I interferons (IFN) by infected cells mediates protection against many viruses, but prolonged or excessive type I IFN secretion can lead to immune pathology. A proper type I IFN response must therefore maintain a balance between protection and excessive IFN secretion. It has been widely noted that the type I IFN response is driven by positive feedback and is heterogeneous, with only a fraction of infected cells upregulating IFN expression even in clonal cell lines, but the functional roles of feedback and heterogeneity in balancing protection and excessive IFN secretion are not clear. To investigate the functional roles for feedback and heterogeneity, we constructed a mathematical model coupling IFN and viral dynamics that extends existing mathematical models by accounting for feedback and heterogeneity. We fit our model to five existing datasets, reflecting different experimental systems. Fitting across datasets allowed us to compare the IFN response across the systems and suggested different signatures of feedback and heterogeneity in the different systems. Further, through numerical experiments, we generated hypotheses of functional roles for IFN feedback and heterogeneity consistent with our mathematical model. We hypothesize an inherent tradeoff in the IFN response: a positive feedback loop prevents excessive IFN secretion, but also makes the IFN response vulnerable to viral antagonism. We hypothesize that cellular heterogeneity of the IFN response functions to protect the feedback loop from viral antagonism. Verification of our hypotheses will require further experimental studies. Our work provides a basis for analyzing the type I IFN response across systems.

## 1. Introduction

Type I interferons (IFN) play a key role in the early immune response to viral infection [1]. The type I IFN response can be divided into two components: IFN induction, involving sensing of virus and consequent transcription and secretion of IFNs, and IFN signaling, involving the binding of extracellular IFN to type I IFN receptors and consequent upregulation of antiviral genes. Type I IFN induction begins when viral molecules are sensed by membrane-bound or cytosolic proteins known as pattern recognition receptors (PRRs). Binding of PRRs to their cognate viral molecules activates cellular pathways, leading to transcription of type I IFN genes and secretion of type I IFNs [2,3,4,5]. Type I IFN signaling begins when extracellular IFN binds to type I IFN receptors, which are found on the surface of almost all cells, leading to activation of the JAK/STAT signaling pathway and upregulation of interferon-stimulated genes (ISGs), a collection of hundreds of genes, many with antiviral properties [6,7]. IFN signaling can occur in an autocrine or paracrine manner, depending on whether a particular IFN protein binds to receptors of the cell that secreted it or other cells, respectively.

Type I IFNs are potent and crucial viral restrictors. IFN-mediated ISGs restrict many viruses [8,9], and IFN deficiencies in mice and humans significantly compromise immune response to viral infections [10]. However, in counterpoint to its key role in restricting viral infection, the type I IFN response is associated with pathology, including auto-immune disease, neuropathology and tissue damage [11,12,13]. The potential harmful effects of type I IFNs are underscored by the existence of extensive negative regulation of IFN pathways and the pathologies that arise when negative regulation is constitutively absent or experimentally blocked [14,15]. A proper IFN response must therefore achieve a balance between protection and excessive IFN secretion. Further, this balance must be achieved in the face of different viruses, which exhibit a range of viral dynamics and a variety of IFN antagonism strategies. For example, HIV-1 has an estimated $R_{0}$ of eight during the first days of infection [16], while New Castle disease virus (NDV) in human cell lines can infect cells, but replicates poorly [17]. Similarly, many viruses encode proteins that antagonize IFN induction and IFN signaling, but the degree and method of IFN antagonism vary greatly across viruses [1,18,19].

The type I IFN response has components that are virus and cellular specific; for example, many ISGs have effector function against particular viruses [9], and cells vary in the pathways that induce IFN [20]. Nevertheless, the IFN response to viral infection has a general form: viruses infect cells; infected cells secrete IFN; and extracellular IFN leads to upregulation of ISGs and protection of cells. At the intercellular level, the functionality of the IFN response is determined by the number of infected cells, the amount of IFN secreted by infected cells and the sensitivity of cellular ISG upregulation to extracellular IFN concentrations. How these factors come together to balance protection and excessive IFN secretion across different viral infections is not well understood.

In particular, two features of the type I IFN response have been noted across many experimental studies: IFN secretion rates are driven by a positive feedback loop and IFN induction is heterogeneous across infected cells. The positive feedback loop is driven by an interplay between IFN induction and signaling. Many of the PRRs and signaling proteins involved in the induction and signaling pathways are themselves ISGs. For example, the proteins IRF7, RIG-I and STAT1 play central roles in the type I IFN pathway and are all ISGs. In fibroblasts and epithelial cells, the type I IFN response typically begins with induction and secretion of IFN-beta, leading to IFN signaling through JAK/STAT, which raises the expression of proteins such as RIG-I, IRF7 and STAT1, which in turn leads to higher levels of IFN-beta induction, as well as IFN-alpha induction [21]. A similar positive feedback loop that is IRF7 dependent exists in dendritic cells [20].

Heterogeneity in cellular IFN expression has been noted in multiple experimental studies, with only a small fraction of infected cells upregulating IFN expression [22,23,24,25,26,27,28,29,30]. In [29], Zhao et al. considered in vitro Sendai virus infection of human and murine fibroblasts. Although almost all fibroblasts were infected, IFN-beta expression was raised above constitutive levels in less than 20% of infected cells. Zhao et al. showed that raising the expression level of RIG-I, TRIM25 and IRF3/7, proteins in the IFN induction pathway, significantly increased the percentage of infected cells that upregulated IFN-beta expression. In [25], Hu et al. demonstrated a similar result using dendritic cells exposed to New Castle disease virus (NDV). Hu et al. found that RIG-I levels were constitutively raised in the subset of dendritic cells that expressed IFN-beta early in infection. More recently, Shalek et al. [28] considered murine dendritic cells exposed to double-stranded RNA and LPS, both of which can stimulate IFN expression. While all cells sensed and altered their gene expression due to RNA or LPS stimulation, only 8% of cells initially showed raised IFN-beta expression.

While positive feedback and heterogeneity in the IFN response have been broadly demonstrated, what functionality these two features have is not clear. A positive feedback loop is thought to restrict a strong IFN response to only significant infections, but what implications does that have on IFN-mediated protection across all infections? What tradeoffs are implicit in the presence of a feedback loop? Previous groups have suggested molecular mechanisms potentially mediating IFN heterogeneity [25,27,31,32,33], but the functional effect of heterogeneity on the type I IFN response has not been studied.

Understanding the functional roles of feedback and heterogeneity benefits from quantitative models of the IFN response. Many authors have constructed intercellular mathematical models of IFN-viral dynamics, allowing for quantitative investigation of the IFN response [34,35,36,37,38,39,40,41,42,43,44,45,46]. All these models, in some way, compartmentalize cells into target cells, infected cells and refractory cells, with refractory cells representing cells that resist viral infection due to ISG expression. Infected and refractory cells are sometimes further split into different compartments, representing different stages of viral infection and innate response, with the exact compartmentalization varying across models. For example, Saenz et al. [46] split infected cells into three compartments—infected cells that have yet to become viral productive, infected cells that have halted infection through ISG upregulation and infected cells that are virally productive—and they split refractory cells into two compartments—cells that are in an initial state of ISG upregulation and can still be infected and cells that are completely resistant to infection (see Figure 2 in Saenz et al.). In contrast, Rand et al. [40] split infected and refractory cells into eight compartmentA (see Figure 6A in Rand et al.). Intercellular models typically also include compartments representing extracellular viral load and IFN concentrations, with interactions between the cellular, viral and IFN compartments described through differential equations.

Notably, most IFN-viral models have not modeled a positive feedback loop and cellular heterogeneity. Further, previous IFN-viral mathematical models have mainly been used to describe and analyze specific systems. For example, Saenz et al. used a mathematical model of equine influenza virus infection to investigate the role of the IFN response in controlling influenza infection. Schmid et al. [41] used a mathematical model of Dengue virus infection to investigate the differing type I IFN response to two Dengue virus strains. Rand et al. used a mathematical model to investigate the stochasticity of the IFN response in the context of New Castle disease virus infection.

In order to investigate the functional roles of positive feedback and heterogeneity in the IFN response, we constructed an intercellular mathematical model of IFN-viral dynamics, similar in structure to existing models, but with the addition of parameters to describe IFN feedback and heterogeneity. We fit the model across five datasets, comparing the fit of the model with and without IFN feedback and estimating the heterogeneity of the IFN response.

Our fits across datasets provided us with a biologically-relevant parameter range over which to investigate IFN-viral dynamics. The parameter range modeled a range of viral infections and IFN secretion rates, allowing us to investigate possible functional roles for a feedback loop and heterogeneity. This portion of our work was based on numerical simulation, and experimental data only entered in defining a proper range for parameter values. Using numerical simulation, we were able to generate hypotheses of functional roles for IFN feedback and heterogeneity. Verification of our hypotheses will require further experimental studies.

## 2. Methods

### 2.1. Model

We constructed a differential equation model of viral-IFN dynamics. The model is presented in detail in the Results section, see model 2 below. Parameters of the model are given in Table 2.

### 2.2. Fitting to Data

We fit model 2 to datasets presented in Rand et al. [40], Patil et al. [47], Saenz et al. [46] and Schmid et al. [41]. For each dataset, we fixed some parameters to dataset authors’ values and fit all other parameters. Fitted parameters are shown in Tables 3–6 below. The fixed parameters are given in the Supplementary Information in Tables S1–S4. All parameters that were fixed were set to the equivalent parameters specified by the dataset authors.

To fit datasets, we used a scaled sum of squares as the negative log likelihood.

$$-\log L\left(\theta \right)=\sum_{j=1}^{D}\sum_{i=1}^{N}{\left(\frac{{\hat{d}}_{j,i}-d_{j,i}\left(\theta \right)}{0.1*max_{i}\left(d_{j,i}\right)}\right)}^{2},$$

where *D* represents the number of data types we considered from the given dataset (e.g., from the Rand et al. dataset, we considered data describing (1) the fraction of cells upregulating IFN-beta mRNA, (2) the fraction of cells upregulating IRF7 mRNA and (3) the extracellular IFN-beta level (U/mL), so D=3), θ is a vector of the parameters we are fitting (e.g., θ=(q,β,ϕ,h,r)), ${\hat{d}}_{j,i}$ is sample *i* from the *j*-th data type, $d_{j.i}\left(\theta \right)$ is our predicted value of $d_{j,i}$ based on numerical simulations of the extended model and $max_{i}\left(d_{j,i}\right)$ is the largest sample value in the *j*-th data type. The 0.1 factor in the denominator reflects an assumption that the standard error is 10% on the scale of the data. We follow Schmid et al., who made the same assumption in constructing their likelihood.

We minimized the negative log likelihood over the fitted parameters using the least_squares function from the Python scipy.optimize package under the default settings. The least_squares function requires initial values for the fitted parameters, off of which the optimization starts. Whenever possible, we used author dataset values as starting values, but when no such values were available, we selected values that gave a reasonable initial fit. We then called least_squares 30 times, perturbing the initial value of each fitted parameter by 10% in an effort to find a global minimum.

### 2.3. Construction of Confidence Intervals and Identifiability

We constructed confidence intervals using likelihood profiles as described in [48]. The likelihood profile for a given parameter is the minimum of the negative log likelihood as a function of the given parameter with all other parameters being allowed to vary:

$$\mathrm{LP}\left(\theta_{i}\right)=\min_{\theta ,\theta_{i}\mathrm{fixed}}\left(-\log L\left(\theta \right)\right),$$

where $\mathrm{LP}(\theta_{i})$ is the likelihood profile and $\theta_{i}$ is the *i*-th coordinate in the θ vector, i.e., the *i*-th fitted parameter. In practice, for each parameter, we selected a grid of values centered around its fitted value and computed the likelihood curve at those grid points. As an example, likelihood curves for each fitted parameter in the Rand et al. dataset are shown in the Supporting Information, see Figures S3 and S4.

Following [48], we assumed:

$$\mathrm{LP}\left(\theta_{i}\right)-\mathrm{LP}\left(\theta_{i}^{*}\right)∼\chi^{2}\left(1\right)$$

where $\theta_{i}^{*}$ is the fitted (optimal) value of $\theta_{i}$ and $\chi^{2}\left(1\right)$ is the chi-squared distribution with 1 degree of freedom. We then calculated 95% confidence intervals as:

$$\left\{\theta_{i}\;|\;\mathrm{LP}\left(\theta_{i}\right)-\mathrm{LP}\left(\theta_{i}^{*}\right)<w_{0.95}\right\}$$

where $w_{0.95}$ is the 0.95 quantile of $\chi^{2}\left(1\right)$, which is roughly 3.85.

In [49], Raue et al. distinguish between three types of parameter identifiability: structural non-identifiability, practical non-identifiability and identifiability. In terms of our computations, structural non-identifiability corresponds to likelihood profiles with no global minimum and represents parameters that cannot be identified even with less noise in the sampling values. Practical non-identifiability corresponds to likelihood profiles with a global minimum, but for which the confidence interval is unbounded because the likelihood curve does not exceed 3.85 and represents parameters that can be precisely identified with less noise in the sampling values. Finally, identifiable parameters have global minimums and finite confidence intervals. The distinction between practical non-identifiable and identifiable parameters depends on the 0.1 standard error that we built into our log likelihood above. Lowering that value would convert the practical non-identifiable parameter to identifiable parameters. Table 1 shows the identifiability of each parameter for each dataset.

### 2.4. Numerical Simulations

Numerical simulations of Model 2 were performed using the odeint function from the Python package PyDDE. For the dataset fits, we set T(0)=1 and all other initial values to 0, except for V(0), which was either fit or set according to dataset author values, as specified above. For the numerical experiments, we set T(0)=0.99 and $E_{1}\left(0\right)=0.01$ with all other initial conditions starting at 0, reflecting an infection at MOI of 0.01.

## 3. Results

**Figure 1:**
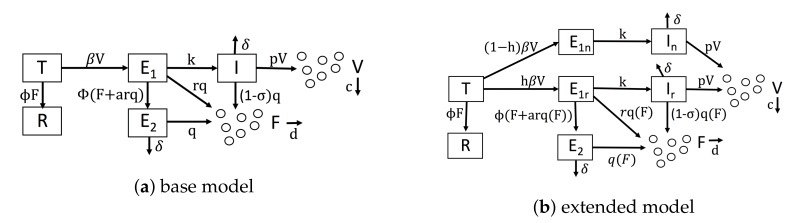
Diagrams of our mathematical models. (**a**) Our base model, which is similar to Saenz et al.’s model. (**b**) Our extended model, which accounts for IFN feedback and heterogeneity. IFN feedback is modeled through the function q(F), which gives the secretion rate as a function of extracellular IFN concentration. Heterogeneity is modeled through the parameter *h*, which gives the fraction of target cells capable of inducing IFN.

As a base model of IFN-viral dynamics, we considered a mathematical model similar to the model of Saenz et al. [46]. Figure 1a shows our baseline model. We modeled uninfected cells through two compartments: target cells, denoted by *T*, which can be infected, and refractory cells, denoted by *R*, which are resistant to viral replication. In many viral infections, when target cells become infected, an initial struggle occurs between the intracellular immune response and the virus for control of the cell [40,50,51,52,53]. For simplicity, we assumed that either the immune system wins and the cell restricts viral replication or the virus wins and the cell becomes virally productive. Accordingly, we modeled infected cells through three compartments: eclipse cells, denoted by $E_{1}$, which are infected cells whose outcome has yet to be determined; effector cells, denoted by $E_{2}$, which are cells that were infected, but have entered an antiviral state that restricts viral replication; and viral cells, denoted by *I*, which are virally productive. Viral cells release virions into the extracellular space, and virions can infect target cells, transforming them into eclipse cells ($E_{1}$). We let *V* be the virion concentration level.

We let *F* be the extracellular IFN concentration. Uninfected cells express and secrete low levels of constitutive IFN, but only infected cells upregulate IFN expression through the IFN induction pathway [2]. We ignored constitutive IFN expression in our model and assumed that IFN is secreted only by infected cells. We assumed that infected cells secrete IFN at a fixed rate, but we allowed the secretion rate to vary depending on the infected cell compartment, i.e., $E_{1}$, $E_{2}$ and *I* cells. IFN has a fast diffusion rate relative to other infection dynamics [54], so many authors have assumed that all secreted IFN immediately diffuses across the extracellular region, e.g., [36,40,41]. Importantly, we modified this assumption by assuming that some secreted IFN is reabsorbed by the secreting cell, a dynamic seen in experiments and theoretical analysis [55,56,57,58,59]. To model this effect, we assumed that for every unit of IFN that is secreted and diffuses into the extracellular region, *a* units of IFN act in an autocrine fashion.

Given these assumptions, we constructed Model 1, which serves as a base model and is defined by the following system of equations.

(1) $$\begin{array}{cc} \dot{T} & =-\beta VT-\phi FT\\ {\dot{E}}_{1} & =\beta VT-kE_{1}-\phi \left(F+arq\right)E_{1}\\ {\dot{E}}_{2} & =\phi \left(F+arq\right)E_{1}-\delta E_{2}\\ \dot{I} & =kE_{1}-\delta I\\ \dot{R} & =\phi FT\\ \dot{F} & =rqE_{1}+qE_{2}+\left(1-\sigma \right)qI-dF\\ \dot{V} & =pI-cV. \end{array}$$

Viruses infect target cells (*T*) and transform them into eclipse cells ($E_{1})$ at rate β. Extracellular IFN transforms target cells into refractory cells (R) at rate ϕ. Eclipse cells are transformed into viral cells (*I*) at rate *k* and are transformed into effector cells ($E_{2}$) at rate ϕF, due to paracrine signaling, and at rate ϕarq, due to autocrine signaling. Effector, eclipse and viral cells secrete IFN at rates *q*, rq and (1−σ)q, respectively. The parameter *r* models lower secretion rates in eclipse cells relative to effector cells due to either delayed IFN induction or viral antagonism. σ models the level of viral antagonism in viral cells on a scale of 0–1, with σ=1 representing complete antagonism. Effector cells die or stop producing IFN, and viral cells die or stop producing virions at rate δ. Viral cells produce virions at rate *p*. Virions and IFN are cleared from the extracellular space at rates *c* and *d*, respectively. Parameter definitions and units are shown in Table 2. Most of our notation is taken from Saenz et al. (compare their Figure 2 to our Figure 1a). Importantly, our base model does not account for IFN feedback or heterogeneity.

**Figure 2:**
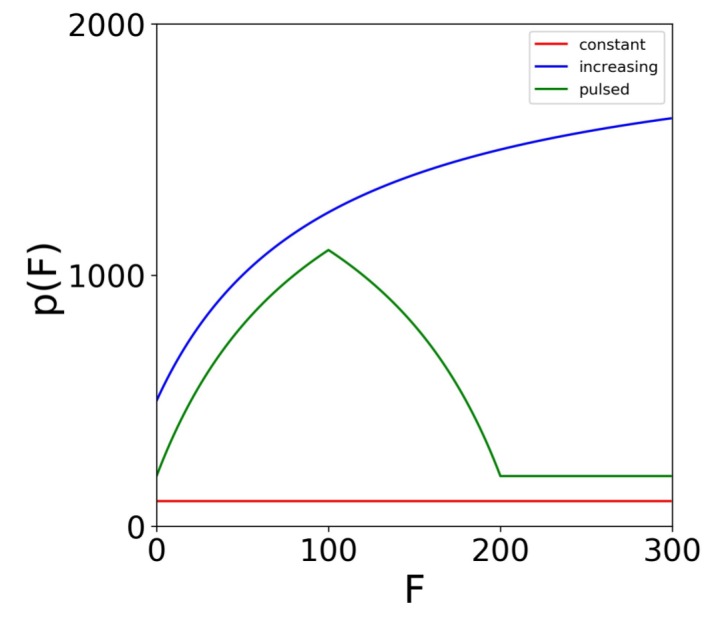
Secretion rate models. Profiles for the constant ($q_{0}=100$), increasing ($q_{0}=500$, $q_{1}=2000$, $\bar{F}=100$) and the pulsed ($q_{0}=200$, $q_{1}=2000$, $\bar{F}=100$) secretion rate models. The models specify the cellular secretion rate (q(F)) as a function of the extracellular IFN level (*F*). For the increasing and pulsed model, $q\left(\bar{F}\right)=\left(q_{0}+q_{1}\right)/2$. For the pulsed model, the maximum secretion rate is achieved at $\bar{F}$.

We extended our base model to account for a positive IFN feedback loop and heterogeneity. To model a feedback loop, we replaced the parameter *q*, which gives the IFN secretion rate of $E_{2}$ cells, by a function q(F) that specifies IFN secretion rates as a function of the extracellular IFN concentration. Intuitively, a feedback loop leads to higher IFN secretion rates as the extracellular IFN concentration *F* rises. We investigated three forms for q(F): a constant rate model parameterized by $q\left(F\right)=q_{0}$, an increasing rate model parameterized by $q\left(F\right)=q_{0}*\bar{F}/\left(\bar{F}+F\right)+q_{1}*F/\left(\bar{F}+F\right)$ where $q_{0}$ is less than $q_{1}$ and a pulsed rate model parameterized by $q\left(F\right)=q_{0}*\bar{F}/\left(\bar{F}+F\right)+q_{1}*F/\left(\bar{F}+F\right)$ when $F<\bar{F}$, $q\left(F\right)=q_{0}*\bar{F}/\left(3\bar{F}-F\right)+q_{1}*\left(2\bar{F}-F\right)/\left(3\bar{F}-F\right)$ when $\bar{F}\leq F\leq 2\bar{F}$ and $q\left(F\right)=q_{0}$ for $F>2\bar{F}$. The parameter $\bar{F}$ is the extracellular IFN concentration at which $q\left(F\right)=\left(q_{0}+q_{1}\right)/2$. For the increasing rate model, the secretion rate starts at $q_{0}$ for F=0 and rises to $q_{1}$ for *F* significantly greater than $\bar{F}$, implicitly modeling a positive IFN feedback loop in which cells secrete more IFN as the extracellular IFN levels rise. The pulsed rate model starts at $q_{0}$ for F=0, rises to $(q_{0}+q_{1})/2$ for $F=\bar{F}$ and then drops and stays at $q_{0}$ as *F* increases further, implicitly modeling a positive feedback loop for $0\leq F\leq \bar{F}$ and a negative feedback loop for $F\geq \bar{F}$. Figure 2 shows the profile of each secretion rate model for particular choices of $q_{0}$, $q_{1}$ and $\bar{F}$.

To model a heterogeneous IFN response, we introduced the parameter *h*, which is the fraction of cells that can induce IFN in response to infection. We split $E_{1}$ (eclipse) cells into two compartments, $E_{1r}$ and $E_{1n}$, reflecting cells that can respond to infection by inducing IFN and those that cannot, respectively, and we similarly split the *I* (viral) cells. Putting our new parameterization of IFN secretion and heterogeneity, we arrive at the following model.

(2) $$\begin{array}{cc} \dot{T} & =-\beta VT-\phi FT\\ {\dot{E}}_{1r} & =h\beta VT-kE_{1r}-\phi \left(F+arq\left(F\right)\right)E_{1r}\\ {\dot{E}}_{1n} & =\left(1-h\right)\beta VT-kE_{1n}\\ {\dot{E}}_{2} & =\phi \left(F+arq\left(F\right)\right)E_{1r}-\delta E_{2}\\ {\dot{I}}_{r} & =kE_{1r}-\delta I_{r}\\ {\dot{I}}_{n} & =kE_{1n}-\delta I_{n}\\ \dot{R} & =\phi FT\\ \dot{F} & =rq\left(F\right)E_{1r}+q\left(F\right)E_{2}+\left(1-\sigma_{I}\right)q\left(F\right)I_{r}-dF\\ \dot{V} & =pI-cV. \end{array}$$

Figure 1b shows the extended model graphically, and Table 2 includes definitions and units for parameters specific to the extended model.

### 3.1. Fitting the Extended Model to Data

We fit our extended model, Model (2), to datasets presented in Rand et al. [40], Patil et al. [47], Saenz et al. [46] and Schmid et al. [41]. For each dataset, we fit our model assuming each of our three secretion models: constant, increasing and pulsed. Results for the increasing rate model were roughly intermediate to the other two, and here, we only present results for the constant and pulsed secretion models. Except for Patil et al., the authors fit their own mathematical intracellular model of IFN-viral dynamics to their dataset. Many of the authors’ models include parameters that connected directly to our own, and in those cases, we used the authors’ inferred values; however, we always obtained our own fit to the parameters describing IFN secretion rates ($q_{0}$, $q_{1}$, $\bar{F}$), IFN induction and signaling rates (*r*, ϕ), viral infectivity rates (β) and the heterogeneity level of IFN induction (*h*). We also fit *p* and σ, except for the Rand et al. and Patil et al. datasets. Those datasets involved New Castle Disease virus infection, for which p=0 and σ=0; see below for details.

Given the parameter values that best fit the data, we constructed a confidence interval for each fitted parameter; see the Methods for details. For Saenz et al., we could not construct meaningful confidence intervals (i.e., confidence intervals other than [0,∞)) for almost all of the parameters, so we set β and ϕ to the values found by Saenz et al., after which we were able to generate meaningful confidence intervals for most of the parameters. Overall, we inferred 5–9 parameters depending on the dataset and secretion rate model.

**Figure 3:**
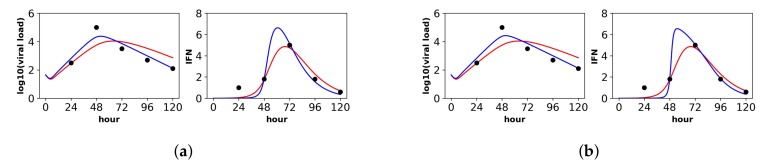
Fit of the extended model to Saenz et al.’s dataset. Shown is the fit of our extended model (blue), assuming a (**a**) constant secretion rate and a (**b**) pulsed secretion rate, and the fit of the Saenz et al. model (red). Our secretion rate models give comparable fits, but the pulsed model suggests faster increases in IFN concentrations.

Below, we discuss each dataset in detail. Overall, the pulsed model was a statistically better fit for the Rand et al. and Schmid et al. datasets. For the Patil et al. and Saenz et al. datasets, the pulsed model led to better, though not statistically significant, fits. In this regard, the fit to the Saenz et al. dataset is particularly instructive. The constant model infers a gradual increase of IFN extracellular concentrations (see the right panel in Figure 3a), while the pulsed model infers a more sudden increase (see the right panel in Figure 3b), but both models fit the data well.

#### 3.1.1. Rand et al.

**Figure 4:**
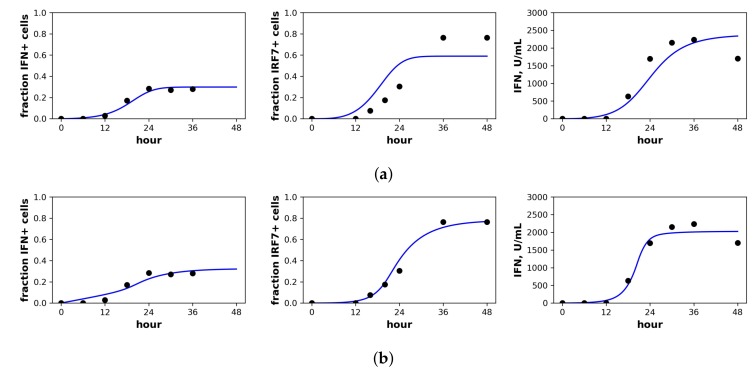
Fit of the extended model to Rand et al.’s dataset. Shown is the fit of our extended model assuming a (**a**) constant secretion rate and (**b**) pulsed secretion rate model. Panels, from left to right, show the fraction of cells expressing IFN mRNA, IRF7 mRNA and the extracellular IFN concentration. The pulsed rate model captures the rapid increase in IFN concentrations between 12 h and 24 h.

Rand et al. studied in vitro New Castle Disease virus (NDV) infection of murine fibroblasts. Importantly, NDV in murine cells does not antagonize the IFN response and cannot produce infection particles, although NDV can enter murine cells and initiate viral protein synthesis [40]. Over the first 48 h of infection, Rand et al. determined the fraction of cells upregulating IFN-beta mRNA, the fraction of cells upregulating IRF7 mRNA, the extracellular IFN-beta level (U/mL) and the total viral expression level of hemagglutinin (HN) across all cells. To connect our model to the data, we assumed that the IFN-beta mRNA level was proportional to cellular IFN secretion rates and that IRF7 was upregulated in our $E_{2}$ and *R* cellular compartments. Since our model does not track viral protein expression and since NDV virions could not initiate second round infections, we did not fit the HN data. Figure 4 shows the fit of our model to the Rand et al. data, and Table 3 provides the fitted parameter values.

For the constant secretion model, we inferred a secretion rate of 1190 U/mL(hour), while for the pulsed secretion rate, we inferred a baseline secretion rate of 100 U/mL(hour), which can rise to 2760 U/mL(hour) when extracellular IFN levels reach 1120 U/mL, which occurred between 18 h and 24 h of infection (Rand et al. estimated a secretion rate of 1300 U/mL(hour), roughly comparable to our values). The pulsed model had a better fit that was statistically significant (*F*-test, *p*-value = $5.6\times 10^{-6}$). Notably, the pulsed model is able to capture the rapid upregulation in IFN concentrations between 12 h and 24 h, while the constant model cannot; compare the far right panels of Figure 4a,b.

We inferred h=0.45 and h=0.66 under constant and pulsed secretion rate models, respectively, suggesting that roughly 45–66% of target cells were capable of inducing IFN upon infection. The confidence intervals for *h* extended to one, but were bounded below by 0.38 and 0.50, so the presence of heterogeneity was not statistically significant, but the absence of extreme heterogeneity was significant. The dataset shows that roughly 30% of cells induce IFN (see fraction IFN+ cells panel in Figure 4). The difference is explained by refractory cells. Our model predicts that roughly half of target cells become refractory. Given 60% of target cells capable of inducing IFN, half become refractory and half become infected, leading to the sampled 30% levels of IFN induction.

#### 3.1.2. Patil et al.

**Figure 5:**
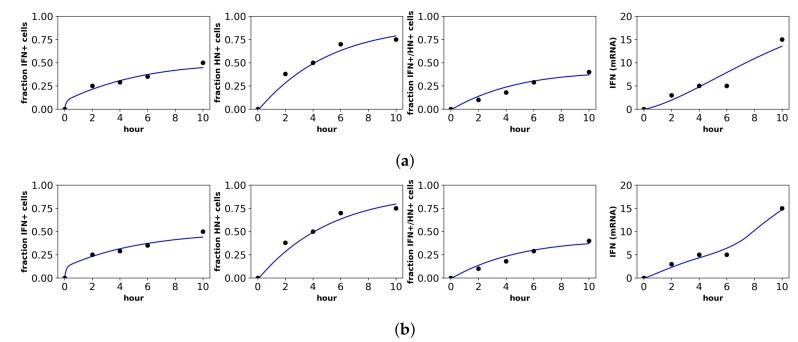
Fit of the extended model to Patil et al.’s dataset. Shown is the fit of our extended model assuming the (**a**) constant secretion rate and (**b**) pulsed secretion rate. Panels, from left to right, show the fraction of cells expressing IFN mRNA, viral HN, both IFN mRNA and viral HN and the average IFN mRNA expression across all cells.

Patil et al. studied in vitro NDV infection of human, monocyte-derived, dendritic cells (DCs). As is the case for the murine cells of Rand et al., NDV does not antagonize IFN expression in human DCs, and NDV infection can lead to viral protein synthesis, but not infectious virions. Patil et al. measured the fraction of cells upregulating IFN-beta mRNA, the fraction of cells expressing viral HN, the fraction of cells upregulating both IFN-beta mRNA and expressing viral HN and the total IFN mRNA expression level across all cells. To connect our model to the data, we assumed that cells upregulating IFN-beta expression were secreting IFN, that only our viral cells (*I* compartment) were expressing viral protein at levels high enough to be detected and that the total IFN mRNA level across all cells was proportional to IFN extracellular concentrations. Figure 5 shows the fit of our models to the Patil et al. data, and Table 4 provides the fitted parameter values.

For both the constant and pulsed models, we could not fit the β parameter. In both cases, fits improved as β increased, suggesting a model in which all cells were immediately infected. Patil et al. reported infection MOIs of roughly one and, based on the presence of viral HN protein, suggested that roughly 75% of cells were infected. Our modeling suggests that all cells were infected, but roughly 25% of cells were either still in the eclipse phase ($E_{1}$ cells) or had become effector cells ($E_{2}$ cells).

Under the constant secretion model, we inferred a secretion rate of 7.3 mRNA/mL(hour), while under the pulsed secretion rate model, we inferred a baseline secretion rate of roughly 9.6 mRNA/mL(hour). The best fits for the parameters $q_{1}$ and $\bar{F}$, 0 and 4.5 mRNA/mL(hour), respectively, suggest downregulation of secretion rates from the baseline secretion rate of 9.6 mRNA/mL(hour). However, our confidence intervals for $q_{1}$ and $\bar{F}$ are wide (indeed, for $\bar{F}$, the confidence interval is unbounded). When we fixed $q_{1}$ to values significantly exceeding $q_{0}$, the best fits pushed $\bar{F}$ to high values, so that IFN secretion rates were never upregulated in the model. Overall, the data is not consistent with a positive feedback loop for IFN secretion rates. We speculate that the short duration of the experiment, 10 h, may not have been long enough to capture positive feedback.

For both constant and pulsed secretion models, we inferred heterogeneity values of roughly 50% with confidence intervals restricted to roughly 45%–55%, matching the results of Patil et al. The pulsed model had a better fit, but the fit was not statistically significant (*F*-test, *p*-value = 0.16).

#### 3.1.3. Saenz et al.

Saenz et al. fit a mathematical model to the dataset of Quinlivan et al. [60] involving in vivo infection by equine influenza A virus (IAV) of horses. Over the course of a week, Quinlivan et al. measured viral load and IFN-alpha levels daily. We limited our analysis to the first five days, after which the NK cell response is thought to shape infection dynamics [39]. Quinlivan et al. measured viral HN levels in nasal secretions and IFN-alpha mRNA levels in blood samples, likely making their measurements reflective of systemic IFN activation.

In contrast to the other datasets, the Saenz et al. dataset is more limited, including only extracellular IFN and virus concentrations and lacking cellular compartment frequencies. As a result, we were not able to identify the parameters β and ϕ for either the constant or pulsed models, meaning that different values of β and ϕ achieved equivalent fits (see the Methods for details). In order to fit our extended model, we therefore used the β and ϕ values inferred by Saenz et al. (see their Table 2). With β and ϕ fixed, we were able to identify the constant rate model parameters, but we still could not identify several of the pulsed rate model parameters. Below, we present the best fit of the data under the pulsed rate model; however, this fit serves simply as a hypothesis consistent with the data, and we cannot assign any statistical certainty to it. Figure 3 shows the fit of our models to the data, and Table 5 provides the fitted parameter values and their confidence intervals (note that several of the pulsed rate model parameters have unbounded confidence intervals, reflecting our inability to identify the parameters).

For the constant secretion rate model, we inferred a secretion rate of 4.7 mRNA/mL(hour). For the pulsed secretion rate model, we inferred a low baseline secretion rate of 0.05, but the secretion rate could be upregulated to roughly 12.5 mRNA/mL(hour) when extracellular IFN levels reached 3.6 mRNA/mL, which occurred at Day 3 of infection. We inferred a heterogeneity level of h=1 for both the constant and pulsed models, meaning that all cells could induce IFN.

Notably, under both the constant and pulsed secretion rate model, we inferred a viral IFN antagonism level of σ=1, meaning complete antagonism of IFN secretion in viral cells (*I* cells). We speculate that our estimates of σ reflect the known antagonism properties of the NS1 protein of influenza [61]. The pulsed model had a better fit, but the better fit was not statistically significant (*F*-test, *p*-value = 0.71).

The parameter *r*, which specifies the IFN secretion rate in eclipse cells relative to effector cells, differed significantly between the two models: 0.00467 and 0.44 for the constant and pulsed models, respectively. Although the difference in *r* estimates is not statistically significant, as seen by the overlap in confidence intervals in Table 5, we do have statistical support that *r* is near zero for the constant model, while we cannot reject r=0.46 for the pulsed model. The difference in these two *r* estimates provides an instructive example of how the two models of secretion rates influence parameter estimates. We find that under the constant model, *r* must be nearly zero to keep IFN concentrations low during the first two days of infection, when many cells are in the eclipse phase. In contrast, under the pulsed model, *r* can be relatively large, i.e., 0.46, because the baseline secretion rate $q_{0}$ is small, allowing IFN concentrations to remain low during the first days of infection, but still rise quickly at later times due to upregulation of IFN secretion rates.

#### 3.1.4. Schmid et al.

**Figure 6:**
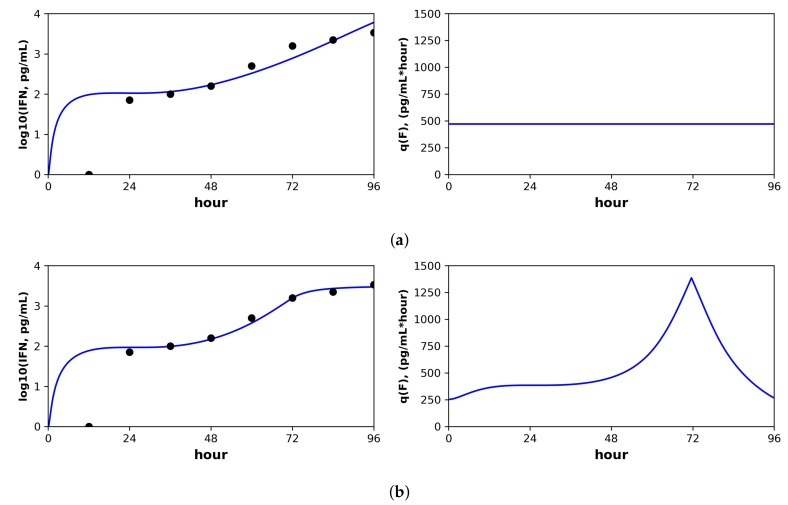
Constant versus pulsed rate secretion model predictions of interferon dynamics for the mutant (MT) dataset of Schmid et al. Shown are the predicted (blue curve) and sampled (black dots) IFN concentration dynamics (left panels) and the predicted IFN secretion rate dynamics (right panels) under our best fit model assuming (**a**) constant secretion rate and (**b**) pulsed secretion rate. Parameter values are shown in Table 6. Fits to the complete Schimd et al. MT dataset are provided in the Supporting Information, see Figures S1 and S2.

Schmid et al. studied in vitro Dengue virus (DENV) infection of A549 cells, a human, lung epithelial, carcinoma cell line that is IFN competent. Two datasets were generated by infecting A549 cells with a DENV wild-type (WT) and mutant (MT) strain, respectively. In each dataset, the number of target, infected and refractory cells and the level of extracellular IFN-lambda (pg/mL) were sampled every 12 h for four days (IFN-lambda is a type III IFN, but Schimd et al. note that type I IFN levels in DENV infection closely track with IFN-lambda). In previous studies, the DENV MT led to attenuated infection relative to the WT, and Schmid et al. sought to understand the mechanistic basis of the attenuation. By fitting a mathematical model to the data, Schmid et al. showed that the MT strain produces more IFN at the early stages of intracellular infection. Table 6 gives the fitted parameter values and confidence intervals. Figure 6 shows the fit of our models to a portion of the Schmid et al. dataset; see the text below for details. Fits of our models to the full Schmid et al. dataset are provided in the Supplementary Information, see Figures S1 and S2.

For both the WT and MT datasets, our inferred baseline IFN secretion rate for the constant rate model was roughly double the pulsed rate model baseline secretion rate (WT: 95 pg/mL(hour) for constant and 58 for pulsed, MT: 470 pg/mL(hour) for constant and 250 for pulsed). Although these differences did not rise to statistical significance, the resultant differences in the predicted IFN dynamics are instructive. Figure 6 shows the predicted IFN concentration and secretion rate dynamics under the constant model (Subplot (a)) and the pulsed model (Subplot (b)) for the MT dataset. The sampled IFN concentrations are also shown. The pulsed model suggests the presence of strong IFN feedback between 48 h and 72 h, when the secretion rates rise from the baseline of 250 pg/mL(hour) to 1500, followed by downregulation back to baseline between hours 72 and 96. The sampled IFN concentrations show an increase between hours 48 and 72 followed by a leveling between hours 72 and 96, dynamics which are well fit by the pulsed rate model. In contrast, the constant rate model cannot capture the leveling between hours 72 and 96. The fit of the pulsed rate model to the full WT and MT datasets was significantly better than the constant rate model (*F*-test, *p*-value: 0.03 (WT) and $7\times 10^{-9}$ (MT)).

Inferred heterogeneity levels suggested a roughly homogeneous response across both WT and MT datasets and both secretion rate models, although our confidence intervals were too broad to provide statistical significance. In contrast, the parameter *r*, which sets the IFN secretion in eclipse cells ($E_{1}$) relative to effector cells ($E_{2}$), differed across the WT and MT datasets. For the WT dataset, *r* was essentially zero for both constant and pulsed rate models, while for the MT dataset, *r* was roughly one for both rate models, with confidence intervals that did not include zero. Our *r* estimates are in line with the results of Schmid et al., suggesting that the MT strain leads to higher levels of IFN transcription at early stages of intracellular infection.

#### 3.1.5. Cross Dataset Comparisons

Given fits to the different datasets, one would like to compare the strength of the IFN responses. However, direct comparison of IFN levels is complicated by differing assays and consideration of different IFNs. Schimd et al. measured human IFN-lambda in pg/mL; Rand et al. measured murine IFN-beta in U/mL, where their U (units) are defined by a murine-specific cellular assay [62]; Saenz et al. measured IFN through comparison of IFN-alpha mRNA levels in blood samples; and Patil et al. measured IFN-beta through mRNA expression levels. Even if we established conversions between the differing assays and IFN types, each dataset involved differing cell types, so that the same amount of IFN may have different effects on ISG upregulation. The same difficulties exist in standardizing viral dynamics.

To circumvent this difficulty, we expressed IFN in units defined so that a single unit of IFN transforms target cells into refractory cells at the rate of one per hour. Put another way, one unit of IFN reduces the frequency of target cells by one log (natural) every hour. Similarly, we expressed viral load in units defined so that a single unit of virions reduces target cell frequency at a rate of one per hour. Biologically, measuring in these units means that we are measuring IFN and virions in terms of their effector function on the given cell types. In contrast, measuring IFN using, for example, international units (IU) corresponds to measuring IFN in terms of its effector function on a standardized cell type and virus [63]. Measuring IFN by density, e.g., pg/mL, standardizes the amount of IFN secreted, but not its effect. With this in mind, we refer to our units as IFN effective units (iEU) and viral effective units (vEU).

Mathematically, measuring in effective units leads to β=1 and ϕ=1, and the only other parameters affected are *p*, which is replaced by p×β, and $q_{0}$, $q_{1}$ and $\bar{F}$, which are replaced by $q_{0}\times \phi ,q_{1}\times \phi$ and $\bar{F}\times \phi$. We can then use *p* as a measure of viral infectivity and the *q* parameters as measures of IFN response strength. Schmid et al. and Rand et al. measured the rate target cells become refractory when exposed to IFN, so for those datasets, we can connect our iEU to standard units. For Rand et al., we estimated ϕ as $2.8\times 10^{-4}$ (constant model) and $0.63\times 10^{-4}$ (pulsed model). Since 1 iEU is 1/ϕ U/mL., 1 iEU is roughly 4000–16,000 U/mL. Rand et al. measured that 100 U/mL of IFN-beta leads to one log drop in target cell frequency in roughly 24 h (see their Figure 5A), so that using their measurements, we would expect 1 iEU to be 2400 U/mL, at the same magnitude, but slightly below our 4000–16,000 U/mL estimate. Similarly, for the Schmid et al. dataset, we estimate ϕ as roughly $10^{-5}$, meaning that 1 iEU would equal $10^{5}$ pg/mL, or in nanograms 100 ng/mL. Schmid et al. found that 10 ng/mL drops target cell frequency by one log after 6 h (see their Figure 7C); using their measurements, 1 EU would equal 60 ng/mL, roughly in line with our estimate of 100 ng/mL. Below, we use EU derived through our fits, but we emphasize that EU can be measured experimentally and can serve to standardize the comparison of IFN response across systems.

In effective units, comparison between datasets is possible. Table 7 shows the *q* parameter values in units of iEU/hour and the *p* parameter in vEU/hour. The $q_{0}$, $q_{1}$ and $\bar{F}$ values are lowest for the Schmid et al. dataset, on the order of 0.001–0.01. For the Patil et al. dataset, the secretion rate values rise to an order of 0.01–0.04, but our confidence intervals cannot rule out lower values. Rand et al.’s secretion rate values are a further order of magnitude higher, 0.07–0.3, and Saenz et al.’ values are even higher, 0.3–1.9. We speculate that the weak IFN response in Schmid et al. reflects viral antagonism of the IFN response, which is presumed absent in the NDV infection of Rand et al., while the Saenz et al. dataset likely reflects systemic infection involving plasmacytoid DCs, leading to high IFN secretion rates. The Patil et al. dataset has relatively low $q_{0}$, $q_{1}$ and $\bar{F}$ values, but this dataset only tracked the first 10 h of infection and may not reflect upregulated IFN secretion rates.

For the extended model, β is the viral infectivity, but in effective units β=1, and we refer to *p* as the infectivity rate since it is a measure of viral replication. In effective units, values for *p* varied from 0.01 for DENV in Schmid et al. to roughly 0.3 for IAV infection in Saenz et al. Since NDV infection does not produce infectious virions, we could not estimate vEU for Rand et al.’s and Patil et al.’s datasets.

### 3.2. Functional Roles for IFN Feedback and Heterogeneity Based on Model Simulations

Using numerical simulations, we investigated potential functional roles for IFN feedback and heterogeneity by considering a range of parameterizations of the IFN response in our extended model. As the IFN response must cope with a range of viral infections, we investigated the relationship between protection and IFN secretion of each particular parameterization of the IFN response across a range of viral parameterizations. In fitting datasets, we found that IFN secretion rates varied roughly on the order of 0.01–1.0 iEU/hour, while the infectivity rate *p* varied between 0.01 and 0.3 vEU/hour. We therefore considered parameterizations roughly within those ranges, although we extended the viral range slightly to 0.5 to account for more virulent viruses than IAV. Below, to place extracellular IFN levels in a more concrete context, we converted our IFN EU to U/mL based on the relation: 1 iEU equals 7500 U/mL (recall, for the Rand et al. dataset we estimated 1 iEU as 4000–16,000 U/mL; here, we pick 7500 within this range). As a touchstone against which to assess whether IFN levels in our simulations below are biologically reasonable, we note that Rand et al. measured maximum extracellular IFN concentration at roughly 2000 U/mL in their in vitro experiment.

#### 3.2.1. Our Model Feedback Loop Mediates Protection without Excessive IFN Secretion

**Figure 7:**
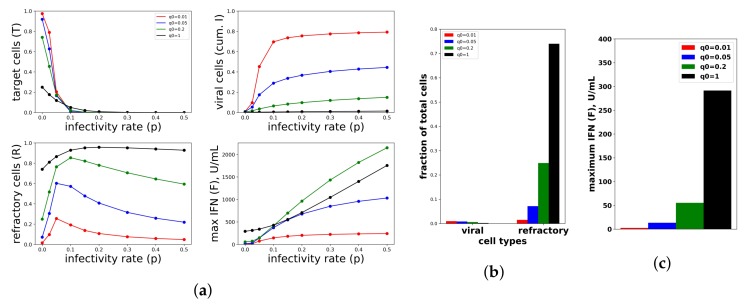
A constant IFN secretion rate cannot simultaneously protect against high infectivity viruses and avoid excessive IFN secretion against low infectivity viruses. (**a**) Shown are the frequency of target cells, *T*, at the end of infection (top left), the fraction of cells that became virally productive, which is the cumulative number of cells in compartment *I* (top right), the total frequency of refractory cells, *R*, at the end of infection (bottom left), and the maximum of the extracellular IFN concentration, *F*, over the course of infection in U/mL (bottom right) as the viral infectivity rate *p* is varied. Each colored curve corresponds to a different $q_{0}$, the IFN secretion rate, as specified in the legend. As *p* increases, protection requires relatively high $q_{0}$ values. Panels (**b**,**c**) are a zoom in of the results in Panel (a) for p=0, representing an abortive infection. As the secretion rate $q_{0}$ varies, the number of virally-productive cells does not change significantly, but the fraction of refractory cells and the maximum IFN concentration levels rise considerably, reflecting excessive IFN secretion. Parameter values not shown: β=1, ϕ=1, a=1, r=0.3, δ=0.05, k=0.1, c=0.3, d=0.15, h=1, σ=0.

To investigate the relationship between protection and IFN secretion for the constant secretion rate model, i.e., $q\left(F\right)=q_{0}$, we considered a range of secretion rates $0\leq q_{0}\leq 1$ over a range of viral infectivity 0≤p≤0.5. Figure 7a shows the frequency of target cells at the end of infection, the fraction of cells that became refractory to infection and virally productive over the course of infection and the maximum extracellular IFN concentration (in U/mL) over the course of infection for the particular values $q_{0}=0.01,0.05,0.2,1.0$ and p=0,0.025,0.05,0.1,0.15,0.2,0.3,0.4,0.5. To generate the figure, we solved Model (2) numerically for each combination of $q_{0},p$. Each point within the panels represents a result from a single simulation. Protection, as quantified by the fraction of cells that become virally productive (with a lower frequency reflecting higher protection), improves across all infectivity rates *p* as the secretion rate $q_{0}$ rises. Functionally, raising secretion rates leads to an increase in the fraction of refractory cells, which leads to the drop in virally-productive cell frequency. Notice that maximum IFN concentrations are on the order of 1000 U/mL for the higher end infectivity rates, in line with the IFN concentrations seen in Rand et al.

The relationship between protection and secretion rates changes markedly when *p* is very small, reflecting a weak infection. Figure 7b shows cellular frequencies and extracellular IFN levels when p=0, an infectivity rate reflecting abortive infection in which the virus can infect cells, but not replicate, e.g., NDV in human cell lines. For all $q_{0}$ values, the fraction of virally infected cells is less than 0.02, but as $q_{0}$ rises, the frequency of refractory cells and extracellular IFN levels rise significantly, with roughly 75% of cells becoming refractory under the highest secretion rate, $q_{0}=1.0$. Since raising secretion rates has little functional effect on restricting infection, we view the IFN secreted and the antiviral cell frequency as measures of excessive IFN secretion. In particular, for $q_{0}=1$, maximum IFN concentrations rose to roughly 300 U/mL in our simulations. In their in vitro study, Rand et al. found that cells exposed to 250 U/mL upregulated their ISGs within 24 h, suggesting that 300 U/mL is a significant IFN level. Intuitively, in low infectivity rate settings, infected cells are largely restricted to cells infected due to an exposure event, and few target cells are subsequently infected. Raising $q_{0}$ raises the amount of IFN produced by these infected cells roughly linearly, leading to a roughly linear increase in the extracellular IFN level and the frequency of antiviral cells, but with little effect on the frequency of infected cells.

**Figure 8:**
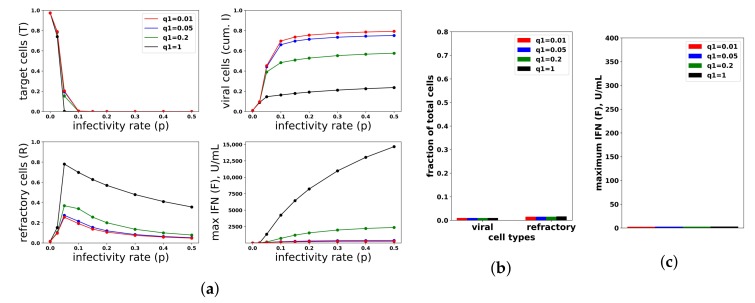
An increasing IFN secretion rate can simultaneously protect against high infectivity viruses and avoid excessive IFN secretion against low infectivity viruses. This figure is analogous to Figure 7, except that an increasing secretion rate is assumed. Each colored curve and bar correspond to a different value of $q_{1}$ (see legend), and we fixed $q_{0}=0.01$. Compare Panels (**b**,**c**) of this figure and Figure 7. See Figure 7 for further details.

To investigate the relationship between protection and IFN secretion rates under an increasing rate model, i.e., $q\left(F\right)=q_{0}*\bar{F}/\left(\bar{F}+F\right)+q_{1}*F/\left(\bar{F}+F\right)$, we considered $q_{1}=0.01,0.05,0.2,1$ and fixed $q_{0}=0.01$. As shown in Figure 8a, raising $q_{1}$ increases protection. As with constant secretion rates, the rise in protection is mediated by an increase in extracellular IFN levels and refractory cell frequency. However, in contrast to the constant secretion rate model, when $q_{1}=1$, extracellular IFN levels reach levels of 15,000 U/mL, far greater than the 2000 U/mL seen in Rand et al.’s in vitro experiments and possibly biologically unrealistic. Figure 8b,c shows the results for the case p=0. Unlike the constant secretion rate, the fraction of cells that become refractory and the extracellular IFN concentration stay low across the different $q_{1}$.

**Figure 9:**
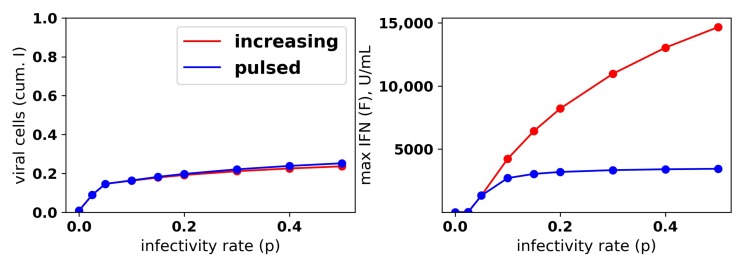
A pulsed IFN secretion rate provides the same protection as an increasing IFN secretion rate, but with lower levels of extracellular IFN. See Figure 8 and the text for details.

We repeated the same numerical experiments assuming a pulsed secretion rate. Infection outcomes were essentially identical as under the increasing secretion rate, except that extracellular IFN concentrations were much lower for a pulsed secretion rate. Figure 9 compares the fraction of virally-infected cells and extracellular IFN levels under increasing and pulsed secretion rate models with $q_{0}=0.01$ and $q_{1}=1$. Strikingly, the fraction of virally-infected cells is roughly identical for both models, but the pulsed secretion rate model produces much less extracellular IFN, on the order of 3000 for the highest infectivity rate p=0.5, in line with the concentration seen by Rand et al.

**Figure 10:**
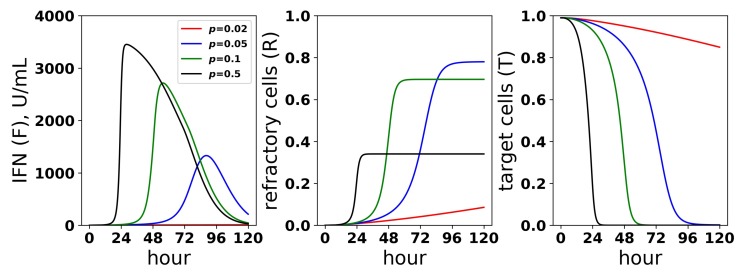
Dynamics of the IFN positive feedback loop. Infection dynamics are shown for different infectivity rates *p*; see the legend. For each *p*, a pulsed secretion rate model was assumed. For p=0.05,0.1,0.5, the feedback loop activates, leading to a rise in extracellular IFN concentrations (F) and a concordant transformation of target cells (T) into refractory cells (R) and the end of infection. For p=0.01, the feedback loop does not activate. Dynamics were simulated using a pulsed secretion rate model with $q_{0}=0.01$, $q_{1}=1$, and $\bar{F}=0.25$.

We sought to better understand how the pulsed rate model mediates protection while avoiding excessive IFN secretion for low infectivity rates *p*. Figure 10 shows the dynamics of extracellular IFN levels and the frequency of refractory and target cells across a range of infectivity rates: p=0.02,0.05,0,1,0.5. For p=0.01, extracellular IFN levels stay low throughout infection, reflecting non-activation of the feedback loop. In contrast, for the higher infectivity rates, extracellular IFN levels experience transitions from low levels to significant levels, reflecting activation of the feedback loop. Concordant with the rise of extracellular IFN levels, target cells are transformed into refractory cells and infection ends. The time span from the beginning of feedback loop activation, which we defined as when extracellular IFN concentration exceeded 50 U/mL—the lowest IFN concentration that Rand et al. found could upregulate ISGs within 48 h in uninfected cells (see their Figure 5A)—to the time of the maximal IFN secretion rate was 34,20 and 9 h for p=0.05,0.1 and 0.5, respectively. Rand et al. noted activation within roughly 24 h (see their Figure 1A), and other authors have noted activation in as little as 4–8 h [25,28].

In summary, a constant secretion rate model is unable to provide protection from high infectivity viruses without excessive IFN secretion against low infectivity viruses. In contrast, a positive feedback loop, modeled by our pulsed secretion rate model, is able to provide protection without excessive IFN secretion. Protection is mediated by activation of the feedback loop, which leads to high levels of extracellular IFN when infectivity rates are high. Excessive IFN secretion is avoided by non-activation when infectivity rates are low.

#### 3.2.2. Our Model Feedback Loop Is Vulnerable to Viral Antagonism

We modeled viral antagonism of IFN induction using the parameters *r* and σ. σ quantifies antagonism in *I* (viral) cells, with zero and one signifying no antagonism and complete antagonism, respectively. The parameter *r* models reduced IFN secretion by $E_{1}$ (eclipse) cells relative to $E_{2}$ (effector) cells, either due to cellular mediated delays in IFN induction or early antagonism of IFN induction by viral proteins.

**Figure 11:**
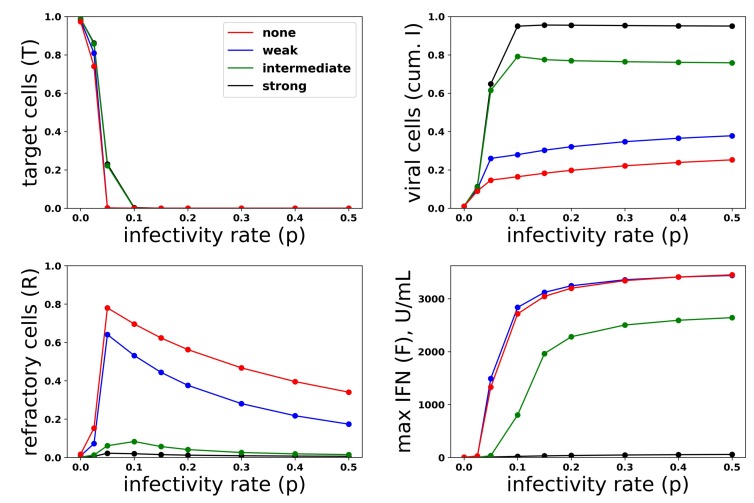
Viral antagonism of IFN induction leads to loss of protection and can block the IFN feedback loop. Colored curves correspond to different levels of viral antagonism of IFN: σ=0, r=0.3 (none), σ=0.5, r=0.2 (weak), σ=0.95, r=0.1 (intermediate) and σ=0.99, r=0.06 (strong). When viral antagonism is strong, extracellular IFN concentration stay low, reflecting blockage of the feedback loop, while for intermediate antagonism maximal, IFN concentrations rise to the significant level, but do not mediate protection. For other levels of antagonism, extracellular IFN concentrations reach high levels, reflecting activation of the feedback loop, and the IFN response mediates significant protection.

Just as we investigated the relationship between protection and IFN secretion across a range of infectivity rates, we investigated the relationship between protection and IFN secretion across a range of antagonism strengths. As an example of strong antagonism, we set σ=0.99 and r=0.06, reflecting 99% and 80% antagonism of virally-productive and eclipse cells, respectively, as compared to our baseline values of σ=0 and r=0.3, which we took as modeling no antagonism. We also considered interpolations between strong and no antagonism: σ=0.95 and r=0.1, corresponding to intermediate antagonism, and σ=0.5 and r=0.2, corresponding to weak antagonism. Figure 11 shows infection outcome for the four cases assuming a pulsed secretion model with $q_{0}=0.01$ and $q_{1}=1$. Strong antagonism blocks the IFN response, with IFN levels only reaching roughly 50 U/mL for the highest infectivity of p=0.5, leading to complete loss of protection. Intermediate antagonism leads to significant maximal IFN concentrations, but the response does not mediate significant protection. For weak and no antagonism, once p>0.1, extracellular IFN levels are nearly maximal, reflecting full activation of the feedback loop, leading to significant levels of protection.

**Figure 12:**
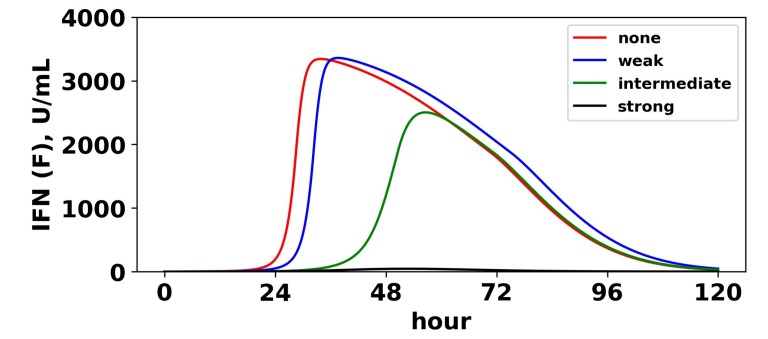
Viral antagonism of IFN induction delays activation of the feedback loop. Extracellular IFN levels (*F*) are shown for four levels of IFN induction antagonism. For all levels, we set p=0.3, and we assumed a pulsed secretion rate model with $q_{0}=0.1$ and $q_{1}=1$. As the level of antagonism is increased, activation of the feedback loop is delayed, and in the case of strong antagonism, the feedback loop is blocked. The delay or blockage of activation leads to the loss of protection, as shown in Figure 11.

To understand the mechanism by which antagonism mediates loss of protection under our model more precisely, we considered extracellular IFN dynamics for the specific case of p=0.3 and a pulsed secretion rate with $q_{0}=0.01$ and $q_{1}=1$. Figure 12 shows the dynamics of extracellular IFN levels. Under strong antagonism, extracellular IFN levels stay low. In contrast, for all other levels of antagonism, the feedback loop activates. However, under intermediate antagonism, the IFN feedback loop activates relatively late and does not reach full activation levels, leading to the loss of protection discussed above. Using 50 U/mL as a mark of IFN activation (as we did above), IFN activation occurs at 20,23 and 33 h and reaches maximal levels within 11,12 and 21 h for none, weak and intermediate antagonism, respectively. The loss of protection under intermediate response can be attributed to the roughly 10-h delay in IFN activation relative to the IFN response under weak antagonism. During those 10 h, 50% of cells become infected, essentially the difference in the fraction of cells that become infected under intermediate antagonism relative to weak antagonism.

In summary, viral antagonism of IFN induction can delay or block feedback loop activation, leading to a loss of protection. Our increasing and pulsed secretion rate models avoid excessive extracellular IFN levels through low secretion rates in early infection, but viral antagonism can exploit the low secretion rates to prevent feedback loop activation.

#### 3.2.3. Cellular Heterogeneity Can Protect Our Model Feedback Loop from Viral Antagonism

Restricting IFN induction to a fraction of cells reduces the average secretion rate per cell, thereby convolving the heterogeneity level with the average secretion rate. To modulate heterogeneity with average secretion rates fixed, we considered a scaled heterogeneity that modulates both the fraction of cells that can induce IFN and the IFN secretion rate of those cells. We assumed that a fraction *h* of cells can induce IFN and that those cells secrete IFN at rate q(F)/h, thereby keeping average secretion per cell fixed at q(F).

**Figure 13:**
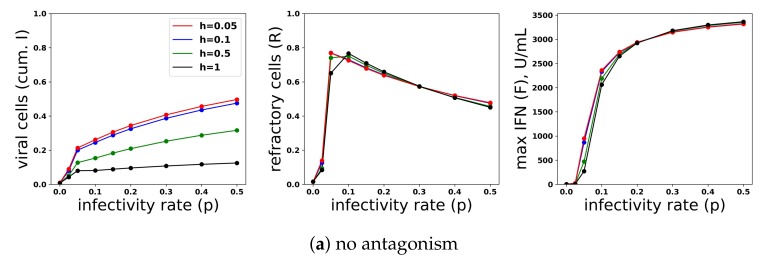

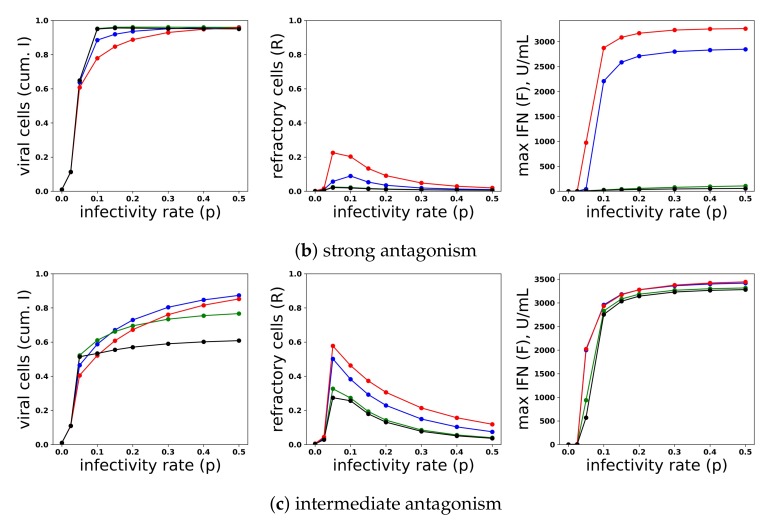
A heterogeneous response provides protection against viral antagonism of IFN induction without excessive IFN secretion for low infectivity viruses. Using model simulations, we compared infection outcome across different levels of heterogeneity for a pulsed secretion rate model. (**a**) When no antagonism is present, infection outcome is similar for different levels of heterogeneity, although the homogeneous response (h=1) provides the best protection. (**b**) Under strong antagonism, the homogeneous IFN response and heterogeneous IFN response at 50% (h=0.5) are blocked, while more heterogeneous responses (h=0.05,0.1) provide protection. (**c**) Under intermediate antagonism, all levels of heterogeneity provide protection, but higher levels of heterogeneity provided better protection.

To investigate the effect of heterogeneity on protection and IFN secretion levels, we considered a pulsed secretion rate $q_{0}=0.1$ and $q_{1}=1$. Figure 13 compares infection outcome over h=0.05,0.1,0.5,1 (h=1 represents a homogeneous response in which all cells can induce IFN), with secretion rates set to q(F)/h. When antagonism is absent (Panel a), the frequency of refractory cells and extracellular IFN concentrations are almost identical across the different values of *h*. The frequency of viral cells increases as *h* drops (i.e., as less cells can induce IFN), but the loss of protection is not extreme. When strong antagonism is present (Panel b), the homogeneous and h=0.5 (50% of cells can induce IFN) IFN responses are blocked, but the heterogeneous responses with h=0.05,0.1 mediate protection, while the IFN feedback loop is activated for all p>0.05. Importantly, regardless of the level of heterogeneity and strength of antagonism, excessive IFN secretion does not occur when infectivity rates are low.

Functionally, under our model, heterogeneity serves to raise the autocrine-mediated IFN signal in eclipse cells while keeping paracrine-mediated signaling in target cells roughly fixed. Eclipse cells are transformed into viral and effector cells at rate *k* and ϕ(F+arq(F)), respectively. The rate of transformation into effector cells can be further decomposed into a paracrine-mediated rate, ϕF, and an autocrine mediated rate ϕarq(F). In early infection, F≈0, and transformation of eclipse cells into effector cells is autocrine mediated and occurs at rate $\phi arq_{0}$. Putting all this together, in early infection, the probability that an eclipse cell becomes an effector cell rather than a viral cell is roughly $\phi arq_{0}/\left(k+\phi arq_{0}\right)$. The presence of antagonism in eclipse cells, parameterized by small values for *r*, lowers this probability to near zero, leading to delay or blockage of feedback loop activation. Our heterogeneity scaling replaces a $q_{0}$ secretion rate by $q_{0}/h$, leading to a higher probability that eclipse cells transform into effector cells. However, solely scaling the secretion rate by $q_{0}/h$ leads to excessive IFN secretion. By restricting IFN induction to a fraction *h* of eclipse cells, heterogeneity functionally creates a small fraction of eclipse cells that are protected by the autocrine response, while maintaining low secretion rates in early infection that keep paracrine signaling in target cells low, thereby avoiding excessive IFN secretion for low infectivity viruses.

**Figure 14:**
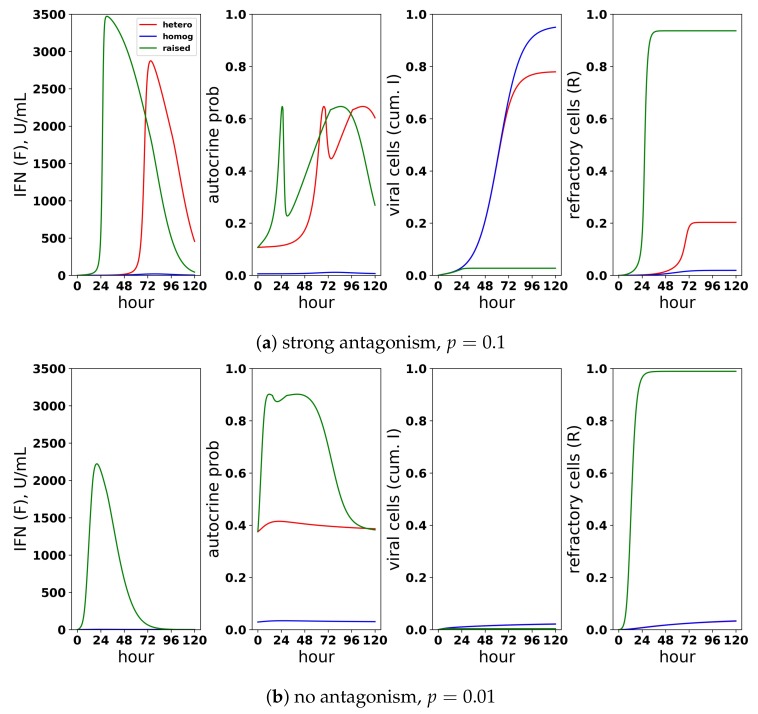
A scaled heterogeneous response protects eclipse cells through autocrine-mediated signaling, while not excessively raising paracrine-mediated signaling to target cells. We considered a scaled heterogeneous response, a homogeneous response and a response in which we raised secretion rates, but did not restrict the fraction of cells that could induce IFN. (**a**) In the presence of strong antagonism and an infectivity rate p=0.1, the feedback loop is activated, and extracellular IFN concentration rises for the heterogeneous and raised responses, but not for the homogeneous response. Rising extracellular IFN levels are mediated by strong autocrine signaling, which leads to a high probability that eclipse cells transform into effector cells (autocrine prob). Activation of the feedback loop leads to a lower frequency of viral cells and a higher frequency of refractory cells, particularly for the raised response. (**b**) In the absence of antagonism and an infectivity rate p=0.01, the heterogeneous and homogeneous feedback loops do not activate, but the feedback loop activates for the raised response. For all three responses, viral cells have low frequency, but the raised response transforms nearly all target cells into refractory cells and raises extracellular IFN levels to 2000 U/mL, reflecting excessive IFN secretion.

Figure 14 shows infection dynamics for three responses: a scaled heterogeneous response with h=0.1 and secretion rates q(F)/h, a homogeneous response (h=1) and a response in which we raised secretion rates to q(F)/h with h=0.1, but assumed that all eclipse cells can induce IFN. The raised secretion rate response serves to demonstrate the importance of scaling both the IFN secretion rate by q(F)/h and restricting IFN secretion to a fraction *h* of eclipse cells. We first investigated the dynamics of these three responses assuming strong antagonism and p=0.1 (Panel a). The heterogeneous and raised responses activate the feedback loop, while the homogeneous feedback loop is blocked. Underlying this difference is autocrine-mediated protection. For the heterogeneous and raised responses, eclipse cells have a 0.20 probability of transforming into effector cells when infection begins, while for the homogeneous response, the probability is ≈0.01. As the feedback loop activates in the heterogeneous and raised responses, autocrine-mediated protection of eclipse cells rises, and eclipse cells have an ≈0.60 probability of transforming into effector cells, while autocrine-mediated protection stays low for the homogeneous response. The result is significantly increased protection relative to the homogeneous response. The raised response activates the feedback loop earlier than the heterogeneous response, leading to higher levels of extracellular IFN and greater protection. We then considered the same three responses assuming no antagonism and p=0.01 (Panel b). In this case, the heterogeneous and homogeneous feedback loops do not activate, while the raised response feedback loop does. There is little difference in protection between the three response, but the raised response transforms almost all target cells into refractory cells, reflecting excessive IFN secretion.

In summary, a scaled heterogeneity raises autocrine-mediated signaling in a fraction of cells that secrete IFN at relatively high rates. Autocrine-mediated signaling protects these cells from relatively high levels of viral antagonism, allowing the cells to transform into effector cells, which can activate the feedback loop. At the same time, the average secretion rate per cell is kept fixed, meaning that extracellular IFN levels remain low in early infection, and excessive IFN secretion does not occur for low infectivity viruses.

## 4. Discussion

To investigate potential functional roles for feedback and heterogeneity, we constructed, fit and analyzed a mathematical model coupling type I IFN and viral dynamics. Our model extends current intercellular-based models by accounting for IFN feedback and heterogeneity. We model IFN feedback by increasing cellular IFN secretion rates as extracellular IFN concentrations rise. We fit our model to several datasets involving different viruses and host cells: in vitro infection of human, epithelial carcinoma cells by mutant and wild-type DENV (Schmid et al. [41]), in vitro infection of murine fibroblasts by NDV (Rand et al. [40]), in vitro infection of human, monocyte-derived dendritic cells by NDV (Patil et al. [47]) and an in vivo infection of ponies by equine IAV (Saenz et al. [46]). We compared fits for our model with and without IFN feedback. We found that inclusion of a feedback loop led to better fits of quick increases in the extracellular IFN concentration, reflecting activation of the feedback loop. In the case of the NDV infection of fibroblasts (Rand et al.) and DENV infection of epithelial carcinoma cells (Schimd et al.), inclusion of the feedback loop led to a statistically significant, better fit. For in vivo IAV infection (Saenz et al.), feedback led to a better, but not statistically significant, fit. Finally, for NDV infection of DCs (Patil et al.), our fits suggested that no feedback was present. We speculate that the short time span of the experiment, 10 h, may have been too short to capture feedback. Our modeling suggests that the quick increase in IFN concentration mediated by a feedback loop can occur over several hours, suggesting that statistical power to identify feedback requires dense temporal sampling.

For NDV infection of fibroblasts and DCs (Rand et al. and Patil et al., respectively), our fits suggested that roughly 50% of cells could induce IFN. For DENV infection of epithelial carcinoma cells and in vivo IAV infection, our fits suggested essentially a homogeneous response, in which all cells induce IFN. We speculate that the homogeneous response reflects systemic infection for the in vivo IAV dataset of Saenz et al. and the noted ability of the DENV mutant to activate the IFN response. Overall, our confidence intervals for *h* were broad, and parameter analysis suggests that accurate inference of *h* requires either relatively low noise in sampling values, denser temporal sampling or sampling of more cellular compartments.

Our current understanding of the IFN response suffers from a lack of cross system analysis, so we sought to compare the IFN response across the fitted datasets. Extracellular IFN concentrations are typically reported as standardized densities, either using weights, e.g., nanograms per mL, or using effector function on some standard assay, e.g., IU/mL [62,63]. We were interested in comparing the strength of the IFN response across datasets. In that context, using standardized units to compare IFN secretion rates assesses which system produced the most IFN. In some contexts, this may be the appropriate measure of strength, but we wanted to measure the ability of the IFN response to mediate protection. Since different cell types respond differently to the same concentrations of IFN, using standardized units does not capture this notion of strength. Instead, we expressed IFN in what we termed effective units, where one effective unit of IFN upregulates ISG expression in one log fraction of target cells. Biologically, effective units can be measured by cellular assay, as done in Rand et al. and Schmid et al.

Using effective units, we noted that the strongest IFN response were seen in in vivo IAV infection (Saenz et al.), while intermediate strength was seen in NDV infection of fibroblasts (Rand et al.), and weak responses were seen in NDV infection of DCs and DENV infection of epithelial cells (Patil et al. and Schmid et al., respectively). For example, our fit of NDV infection of fibroblasts suggested that peak upregulation of the feedback loop led to IFN secretion rates of 0.15 effective units per hour, which corresponds to protection of roughly 15% of target cells every hour. In contrast, our fit of DENV infection of epithelial cells suggested peak extracellular IFN secretion rates of 0.005, which corresponds to protection of roughly 1% of target cells per hour.

The notion of effective units also allowed us to combine our fits to define a biologically meaningful parameter range for IFN secretion and viral infectivity rates. Using this range, we investigated potential functional roles for feedback and heterogeneity through numerical simulations. We found that a relatively high constant secretion rate leads to excessive IFN secretion in abortive viral infections, while a relatively low constant secretion rate cannot protect against high infectivity viruses. In contrast, a feedback loop was able to avoid this tradeoff by setting a low baseline secretion rate that rises only under relatively high viral infectivity rates. Further numerical simulations showed that modulation of secretion rates by a feedback loop creates a vulnerability that can be exploited by viral antagonism. To avoid excessive IFN secretion, a feedback loop must keep secretion rates low in early infection, but this essential feature is vulnerable to viral antagonism. When secretion rates are low, viral antagonism of IFN induction can keep extracellular IFN levels from rising to levels that activate the feedback loop, leading to a delay or blockage in activation of the feedback loop. Putting all this together, based on our numerical simulations, we hypothesize that the IFN feedback loop balances protection and excessive IFN secretion, but with the price of vulnerability to viral antagonism.

We next used numerical simulations to consider a functional role for heterogeneity. We modeled a scaled heterogeneity, in which a fraction *h* of infected cells can induce IFN with secretion rates that are raised by a factor 1/h. The higher secretion rates in this subset of infected cells increases autocrine-mediated IFN signaling, thereby making it more likely that these cells will upregulate their ISGs and become effector cells, which secrete IFN free from viral antagonism. On the other hand, restricting IFN induction to a fraction *h* of cells keeps the average secretion rate per infected cell fixed, which preserves the extracellular IFN levels mediated by the feedback loop. Putting these two features together, based on our numerical simulations, we hypothesize that heterogeneity protects the feedback loop from viral antagonism through autocrine-mediated IFN signaling in the fraction of cells capable of inducing IFN.

Work by Hu et al. [25] and Shalek et al. [28] suggests that a small fraction of cells, termed initiator cells, are responsible for activating the IFN response. In particular, Shalek et al. experimentally demonstrated, in an in vitro system, that stimulation of dendritic cells by PRR agonists led to high levels of IFN secretion by a small fraction of cells. When autocrine-mediated IFN signaling was blocked, this response was lost. Our modeling suggests that the functional role of limiting IFN induction to initiator cells is to provide a protective autocrine response without raising extracellular IFN levels to excessive levels.

While we are not aware of previous modeling analysis of the effects of heterogeneity on infection outcome, several authors have suggested possible benefits associated with a heterogeneous response. Rand et al. demonstrated heterogeneity in the IFN-beta response time of infected cells and suggested that this heterogeneity may allow for a sustained IFN response [40]. Our modeling suggests that IFN mediated protection is dependent on activation of the feedback loop, rather than sustaining an IFN response, but it is possible that protection against viruses with extended intracellular times may require a sustained response. Zhao et al. suggested that heterogeneity may allow cells to achieve an optimal level of extracellular type I IFN by modulating the frequency of expressing cells [29]. Our modeling supports exactly such a view. Ivashkiv and Donlin and, more recently, Czerkies et al. suggested that heterogeneity may allow for a small fraction of infected cells to mass produce IFN and then enter apoptosis [30,64]. Such mass producers would protect other cells through paracrine signaling and prevent further viral replication through their own apoptosis. While our modeling also suggests that mass producers of IFN protect other cells through paracrine signaling, we suggest that mass producers are protected by autocrine signaling, and we do not model apoptosis. Czerkies et al. noted apoptosis in the case of fibroblasts exposed to RIG-I and MDA5 agonists, but whether this would also be the case under viral infection for mass producers is unclear. Further, the extent to which cells exposed to ongoing viral replication are able to secrete IFN is dependent on virus and cell types [65]. It may be that in some cases, mass producers are protected, while in other cases, apoptosis results.

Given the complexity of the type I IFN response and viral infection, our modeling has many limitations. We have not distinguished between the different type I IFNs, in particular IFN-β and IFN-α, although both are crucial to the IFN response and are induced with different dynamics and by different cell types [20,66]. We have not considered spatial effects. In particular, we modeled the IFN-response as either autocrine or paracrine, but in reality, IFN secreted by a cell is more likely to bind to IFN receptors of nearby cells, a phenomenon observed in experimental studies [26,56,67]. We have also not considered the interaction between IFN and other early cytokines such as IL-1, e.g., [68,69]

On the other hand, our results have some general features that transcend our model. Under our model, low secretion rates in early infection leave the feedback loop vulnerable to antagonism, and heterogeneity makes the feedback loop more robust by increasing autocrine-mediated protection. Regardless of the model, if the IFN response is driven by a positive feedback loop, then there must be cases when the feedback loop is not activated in response to infection, otherwise excessive extracellular IFN levels would arise in response to abortive infections. If activation of the feedback loop is largely driven by extracellular IFN levels, then our results would likely still hold since viral antagonism can modulate extracellular IFN levels and therefore modulate feedback loop activation. However, the extent to which IFN induction and signaling within a specific cell is dependent on extracellular IFN levels is unclear. IFN independent pathways that upregulate ISGs exist [70,71]. Individual cells can alter their IFN secretion rates through autocrine-mediated IFN signaling, an effect that is not present in our model, which assumes that secretion rates are determined by a single global extracellular IFN level. Cells are also sensitive to signals of ongoing infection, for example the presence of defective interfering viral particles [72,73], which serve to activate the IFN response independent of extracellular IFN levels. Further work is needed to investigate such effects and their impact on the IFN response.

## Figures and Tables

**Table 1:** Identifiability characteristics of the fitted parameters. We categorize each fitted parameter as structural non-identifiable (structural), practically non-identifiable (practical) or identifiable.

	Constant Rate Model			Pulsed Rate Model		
Dataset	Structural	Practical	Identifiable	Structural	Practical	Identifiable
Rand et al.		*h*	$q_{0},\beta ,\phi ,r$		$q_{0}$, $q_{1}$, $\bar{F},h$	β,ϕ,r
Saenz et al. ^#^			$q_{0},p,h,\sigma$	$q_{0},r,\sigma$	$q_{1},\bar{F}$	p,h
Patil et al.	β	ϕ,r	$q_{0},k,h$	β	$q_{0},q_{1},\bar{F},k$	ϕ,r,h
Schmid et al. (MT)		$\sigma ,q_{0},h$	β,ϕ,r,p	r,h	$q_{0},q_{1},\bar{F},\sigma$	β,ϕ,p
Schmid et al. (WT)		$q_{0},h$	β,ϕ,r,p,σ		$q_{0},q_{1},r,h,\sigma$	$\bar{F},\beta ,\phi ,p$

^#^ = for Saenz et al., we fixed β and ϕ.

**Table 2:** Model parameters Units for IFN varied between datasets: Schmid et al. (IFN-lambda, pg/mL), Rand et al. (IFN-beta, U/mL), Saenz et al. (IFN-alpha, mRNA/mL), Patil et al. [47] (IFN-beta, mRNA/mL).

Parameter	Symbol	Units
baseline IFN secretion rate	$q_{0}$	IFN/mL(hour)
upregulation IFN secretion rate	$q_{1}$	IFN/mL(hour)
IFN upregulation constant	$\bar{F}$	IFN/mL
viral infectivity rate	β	mL/virus(hour)
IFN protection rate	ϕ	mL/IFN(hour)
autocrine IFN fraction	*a*	
eclipse cell IFN secretion fraction	*r*	
viral and effector cell clearance rate	δ	1/h
eclipse to viral cell transformation rate	*k*	1/h
virion production rate	*p*	virus/hour
virion clearance rate	*c*	1/
extracellular IFN clearance rate	*d*	1/h
fraction of target cells that can induce IFN	*h*	
viral antagonism level	σ	

**Table 3:** Fitted parameters for Rand et al.’s dataset under constant and pulsed secretion rate models. Parameters not shown were set to values determined in Rand et al; see Table S1 in the Supporting Information. SSE is the sum of squared errors. Confidence intervals are at 95% level. IFN units are U/mL, and viral load units are HN/mL; see Table 2 for the units of each parameter.

Parameter	Constant	Pulsed
$q_{0}$	1190 [1030,1390]	100 [30,570]
$q_{1}$		5420 [3620,∞)
$\bar{F}$		1120 [990,1320]
β	$1.5\times 10^{-3}$ [$0.30\times 10^{-3}$, $2.0\times 10^{-3}$]	$0.65\times 10^{-3}$ [$0.34\times 10^{-3}$, $1.0\times 10^{-3}$]
ϕ	$2.8\times 10^{-4}$ [$0.26\times 10^{-4}$, $3.8\times 10^{-4}$]	$0.63\times 10^{-4}$ [$0.31\times 10^{-4}$, $1.6\times 10^{-4}$]
*r*	0.00403 [0,0.88]	0.37 [0.14,0.68]
*h*	0.45 [0.38,1]	0.66 [0.50,1]
(SSE)	49.27	17.99

**Table 4:** Fitted parameters for Patil et al.’s dataset under constant and pulsed secretion rate models. Confidence intervals are at the 95% level. IFN and viral units are in terms of mRNA/mL.

Parameter	Constant	Pulsed
$q_{0}$	7.3 [6,9]	9.6 [2,13]
$q_{1}$		0 [0,7]
$\bar{F}$		4.5 [1.5,∞]
β	∞ [0.70,∞]	∞ [0.78,∞]
ϕ	0.0059 [0,0.026]	0.0047 [0,0.025]
*k*	0.18 [0.14,∞]	0.17 [0.15,∞]
*r*	0.19 [0,1]	0.25 [0.04,1]
*h*	0.49 [0.44,0.56]	0.48 [0.42,0.56]
(SSE)	13.63	9.50

**Table 5:** Fitted parameters for the Saenz et al. dataset under constant and pulsed secretion rate models. Confidence intervals are at the 95% level. IFN and viral units are in units of mRNA/mL.

Parameter	Constant	Pulsed
$q_{0}$	4.7 [3.2,18.6]	0.05 [0.05,∞]
$q_{1}$		12.6 [0,∞]
$\bar{F}$		3.6 [0,∞]
*r*	0.00467 [0,0.02]	0.46 [0,1]
*p*	44,800 [308,00,63,100]	45,400 [0,69,000]
*h*	1 [0.54,1]	1 [0.58,1]
σ	1 [0.90,1]	1 [0,1]
(SSE)	3.67	3.10

**Table 6:** Fitted parameters for the Schmid et al. wild-type and mutant datasets under constant and pulsed secretion rate models. IFN and viral units are in terms of pg/mL and a.u./mL, respectively.

Parameter	Wild-Type		Mutant	
	Constant	Pulsed	Constant	Pulsed
$q_{0}$	95 [70,∞]	58 [40,∞]	470 [300,∞]	250 [100,∞]
$q_{1}$		810 [200,∞]		2520 [970,∞]
$\bar{F}$		95 [39,170]		1490 [100,2420]
$\beta {\;}^{\#}$	1.5 [1.0,2.2]	0.6 [0.3,1.1]	1.2 [0.73,1.9]	1.7 [0.9,3.3]
$\phi {\;}^{\#}$	1.1 [0.8,1.7]	1.1 [0.7,1.8]	0.9 [0.5,1.5]	1.0 [0.6,1.5]
*r*	0 [0,0.36]	0.00172 [0,1]	1 [0.12,1]	1 [0.06,1]
*p*	840 [450,1390]	3000 [1280,11,020]	580 [280,1040]	340 [100,880]
*h*	1 [0.02,1]	1 [0.02,1]	1 [0.02,1]	0.83 [0.02,1]
σ	0 [0,0.68]	0.02 [0,1]	1 [0,1]	1 [0,1]
(SSE)	43.00	26.94	44.90	34.96

^#^ All values for β and ϕ are in units of $10^{-5}$.

**Table 7:** Parameters for datasets in effective units. Effective units allow for comparison of the IFN response across datasets. We define IFN effective units (iEU) and viral effective units (vEU) as the extracellular IFN concentration and virion concentration, respectively, necessary to reduce target cell frequency by 1 natural log during 1 h. Units are vEU/hour (*p*), iEU/hour ($q_{0}$ and $q_{1}$) and iEU ($\bar{F}$).

	**Schmid WT**		**Schmid MT**			
	**Constant**	**Pulsed**	**Constant**	**Pulsed**		
*p*	0.01 [0.01,0.02]	0.02 [0.02,0.03]	0.007 [0.005,0.01]	0.006 [0.004,0.009]		
$q_{0}$	0.001 [0.0008,0.001]	0.0006 [0.0004,0.0009]	0.004 [0.003,0.006]	0.003 [0.002,0.004]		
$q_{1}$		0.009 [0.006,0.01]		0.025 [0.012,0.046]		
$\bar{F}$		0.001 [0.0009,0.001]		0.015 [0.008,0.022]		
	**Rand**		**Saenz**		**Patil**	
	**Constant**	**Pulsed**	**Constant**	**Pulsed**	**Constant**	**Pulsed**
*p*			0.3 [0.2,0.4]	0.3 [0.2,0.5]		
$q_{0}$	0.3 [0.03,0.4]	0.006 [0.002,0.01]	0.7 [0.5,2.9]	0.008 [0.00006,0.7]	0.04 [0.00004,0.19]	0.04 [0.0004,0.23]
$q_{1}$		0.3 [0.2,0.8]		1.9 [1.4,32.3]		0 [0,0]
$\bar{F}$		0.07 [0.03,0.19]		0.56 [0,3.1]		0.025 [.00002,0.11]

## Supplementary Materials

The following are available online at http://www.mdpi.com/1999-4915/10/10/517/s1.

## References

[1] Randall R.E., Goodbourn S. (2008). Interferons and viruses: An interplay between induction, signalling, antiviral responses and virus countermeasures. J. Gen. Virol..

[2] Takeuchi O., Akira S. (2010). Pattern Recognition Receptors and Inflammation. Cell.

[3] Loo Y.M., Gale M. (2011). Review Immune Signaling by RIG-I-like Receptors. Immunity.

[4] Sparrer K.M.J., Gack M.U. (2015). Intracellular detection of viral nucleic acids. Curr. Opin. Microbiol..

[5] Kawai T., Akira S. (2010). The role of pattern-recognition receptors in innate immunity: Update on toll-like receptors. Nat. Immunol..

[6] Stark G.R., Darnell J.E. (2012). The JAK-STAT Pathway at Twenty. Immunity.

[7] Schneider W.M., Chevillotte M.D., Rice C.M. (2014). Interferon-stimulated genes: A complex web of host defenses. Annu. Rev. Immunol..

[8] Schoggins J.W., Wilson S.J., Panis M., Murphy M.Y., Jones C.T., Bieniasz P., Rice C.M. (2011). A diverse range of gene products are effectors of the type I interferon antiviral response. Nature.

[9] Schoggins J.W., Rice C.M. (2011). Interferon-stimulated genes and their antiviral effector functions. Curr. Opin. Virol..

[10] Levy D.E. (1999). Physiological significance of STAT proteins: Investigations through gene disruption in vivo. Cell. Mol. Life Sci..

[11] Trinchieri G. (2010). Type I interferon: friend or foe?. J. Exp. Med..

[12] Rodero M.P., Crow Y.J. (2016). Type I interferon–mediated monogenic autoinflammation: The type I interferonopathies, a conceptual overview. J. Exp. Med..

[13] Hofer M.J., Campbell I.L. (2016). Immunoinflammatory diseases of the central nervous system—The tale of two cytokines. Br. J. Pharmacol..

[14] Linossi E.M., Babon J.J., Hilton D.J., Nicholson S.E. (2013). Suppression of cytokine signaling: The SOCS perspective. Cytokine Growth Factor Rev..

[15] Rice G.I., Del Toro Duany Y., Jenkinson E.M., Forte G.M., Anderson B.H., Ariaudo G., Bader-Meunier B., Baildam E.M., Battini R., Beresford M.W. (2014). Gain-of-function mutations in IFIH1 cause a spectrum of human disease phenotypes associated with upregulated type i interferon signaling. Nat. Genet..

[16] Ribeiro R.M., Qin L., Chavez L.L., Li D., Self S.G., Perelson A.S. (2010). Estimation of the initial viral growth rate and basic reproductive number during acute HIV-1 infection. J. Virol..

[17] Park M.A., García-sastre A., Cros J.F., Basler C.F., Palese P. (2003). Newcastle Disease Virus V Protein Is a Determinant of Host Range Restriction Newcastle Disease Virus V Protein Is a Determinant of Host Range Restriction. J. Virol..

[18] García-Sastre A. (2017). Ten Strategies of Interferon Evasion by Viruses. Cell Host Microbe.

[19] Haller O., Kochs G., Weber F. (2006). The interferon response circuit: Induction and suppression by pathogenic viruses. Virology.

[20] Honda K., Yanai H., Negishi H., Asagiri M., Sato M., Mizutani T., Shimada N., Ohba Y., Takaoka A., Yoshida N. (2005). IRF-7 is the master regulator of. Nature.

[21] Levy D.E., Marié I., Smith E., Prakash A. (2002). Enhancement and diversification of IFN induction by IRF-7-mediated positive feedback. J. Interferon Cytokine Res..

[22] Zawatzky R., De Maeyer E., De Maeyer-Guignard J. (1985). Identification of individual interferon-producing cells by in situ hybridization. Proc. Natl. Acad. Sci. USA.

[23] Enoch T., Zinn K.A.I., Maniatis T.O.M. (1986). Activation of the Human Inducible Factor P-Interferon Gene Requires an Interferon-Inducible Factor. J. Virol..

[24] Apostolou E., Thanos D. (2008). Virus Infection Induces NF-*κ*B-Dependent Interchromosomal Associations Mediating Monoallelic IFN-*β* Gene Expression. Cell.

[25] Hu J., Nudelman G., Shimoni Y., Kumar M., Ding Y., López C., Hayot F., Wetmur J.G., Sealfon S.C. (2011). Role of cell-to-cell variability in activating a positive feedback antiviral response in human dendritic cells. PLoS ONE.

[26] Chen S., Short J.A.L., Young D.F., Killip M.J., Schneider M., Goodbourn S., Randall R.E. (2010). Heterocellular induction of interferon by negative-sense RNA viruses. Virology.

[27] Hwang S.Y., Hur K.Y., Kim J.R., Cho K.H., Kim S.H., Yoo J.Y. (2013). Biphasic RLR-IFN-*β* response controls the balance between antiviral immunity and cell damage. J. Immunol..

[28] Shalek A.K., Satija R., Shuga J., Trombetta J.J., Gennert D., Lu D., Chen P., Gertner R.S., Gaublomme J.T., Yosef N. (2014). Single-cell RNA-seq reveals dynamic paracrine control of cellular variation. Nature.

[29] Zhao M., Zhang J., Phatnani H., Scheu S., Maniatis T. (2012). Stochastic Expression of the Interferon-*β* Gene. PLoS Biol..

[30] Czerkies M., Korwek Z., Prus W., Kochańczyk M., Jaruszewicz-Błońska J., Tudelska K., Błoński S., Kimmel M., Brasier A.R., Lipniacki T. (2018). Cell fate in antiviral response arises in the crosstalk of IRF, NF-*κ*B and JAK/STAT pathways. Nat. Commun..

[31] Cai C., Zhou J., Sun X., Sun T., Xie W., Cui J. (2017). Integrated modeling and analysis of intracellular and intercellular mechanisms in shaping the interferon response to viral infection. PloS ONE.

[32] Zhang W., Tian T., Zou X. (2015). Negative feedback contributes to the stochastic expression of the interferon gene in virus-triggered type I interferon signaling pathways. Math. Biosci..

[33] Levin D., Harari D., Schreiber G. (2011). Stochastic Receptor Expression Determines Cell Fate upon Interferon Treatment. Mol. Cell. Biol..

[34] Bazhan S.I., Belova O.E. (1999). Interferon-induced antiviral resistance. A mathematical model of regulation of Mx1 protein induction and action. J. Theor. Biol..

[35] Fribourg M., Hartmann B., Schmolke M., Marjanovic N., Albrecht R.A., García-Sastre A., Sealfon S.C., Jayaprakash C., Hayot F. (2014). Model of influenza A virus infection: Dynamics of viral antagonism and innate immune response. J. Theor. Biol..

[36] Getto P., Kimmel M., Marciniak-Czochra A. (2008). Modelling and analysis of dynamics of viral infection of cells and of interferon resistance. J. Math. Anal. Appl..

[37] Howat T.J., Barreca C., O’Hare P., Gog J.R., Grenfell B.T. (2006). Modelling dynamics of the type I interferon response to in vitro viral infection. J. R. Soc. Interface.

[38] Miao H.Y., Hollenbaugh J.A., Zand M.S., Holden-Wiltse J., Mosmann T.R., Perelson A.S., Wu H.L., Topham D.J. (2010). Quantifying the Early Immune Response and Adaptive Immune Response Kinetics in Mice Infected with Influenza A Virus. J. Virol..

[39] Pawelek K.A., Huynh G.T., Quinlivan M., Cullinane A., Rong L., Perelson A.S. (2012). Modeling within-host dynamics of influenza virus infection including immune responses. PLoS Comput. Biol..

[40] Rand U., Rinas M., Schwerk J., Nöhren G., Linnes M., Kröger A., Flossdorf M., Kály-Kullai K., Hauser H., Höfer T. (2012). Multi-layered stochasticity and paracrine signal propagation shape the type-I interferon response. Mol. Syst. Biol..

[41] Schmid B., Rinas M., Ruggieri A., Acosta E.G., Bartenschlager M., Reuter A., Fischl W., Harder N., Bergeest J.P., Flossdorf M. (2015). Live Cell Analysis and Mathematical Modeling Identify Determinants of Attenuation of Dengue Virus 2^′^-*O*-Methylation Mutant. PLoS Pathog..

[42] Seto J., Qiao L., Guenzel C.A., Xiao S., Shaw M.L., Hayot F., Sealfon S.C. (2010). Novel Nipah Virus Immune-Antagonism Strategy Revealed by Experimental and Computational Study. J. Virol..

[43] You L., Yin J. (1999). Amplification and spread of viruses in a growing plaque. J. Theor. Biol..

[44] Zaslavsky E., Hayot F., Sealfon S.C. (2012). Computational approaches to understanding dendritic cell responses to influenza virus infection. Immunol. Res..

[45] Zou X., Xiang X., Chen Y., Peng T., Luo X., Pan Z. (2010). Understanding inhibition of viral proteins on type I IFN signaling pathways with modeling and optimization. J. Theor. Biol..

[46] Saenz R.A., Quinlivan M., Elton D., MacRae S., Blunden A.S., Mumford J.A., Daly J.M., Digard P., Cullinane A., Grenfell B.T. (2010). Dynamics of Influenza Virus Infection and Pathology. J. Virol..

[47] Patil S., Fribourg M., Ge Y., Batish M., Tyagi S., Hayot F., Sealfon S.C. (2015). Single-cell analysis shows that paracrine signaling by first responder cells shapes the interferon-*β* response to viral infection. Sci. Signal..

[48] Venzon A.D.J., Moolgavkar S.H. (1988). A Method for Computing Profile-Likelihood-Based Confidence Intervals. J. R. Stat. Soc. Ser. C.

[49] Raue A., Kreutz C., Maiwald T., Bachmann J., Schilling M., Klingmüller U., Timmer J. (2009). Structural and practical identifiability analysis of partially observed dynamical models by exploiting the profile likelihood. Bioinformatics.

[50] Davis M.E., Wang M.K., Rennick L.J., Full F., Gableske S., Mesman A.W., Gringhuis S.I., Geijtenbeek T.B.H., Duprex W.P., Gack M.U. (2014). Antagonism of the phosphatase PP1 by the measles virus v protein is required for innate immune escape of MDA5. Cell Host Microbe.

[51] Andrejeva J., Young D.F., Goodbourn S., Randall R.E. (2002). Degradation of STAT1 and STAT2 by the V Proteins of Simian Virus 5 and Human Parainfluenza Virus Type 2, respectively: Consequences for Virus Replication in the Presence of Alpha/Beta and Gamma Interferons Degradation of STAT1 and STAT2 by the V Prote. J. Virol..

[52] Valmas C., Basler C.F. (2011). Marburg Virus VP40 Antagonizes Interferon Signaling in a Species-Specific Manner. J. Virol..

[53] Westcott M.M., Ahmed M., Smedberg J.R., Rajani K.R., Hiltbold E.M., Lyles D.S. (2013). Preservation of dendritic cell function during vesicular stomatitis virus infection reflects both intrinsic and acquired mechanisms of resistance to suppression of host gene expression by viral M protein. J. Virol..

[54] Kreuz L.E., Levy A.H. (1965). Physical Properties of Chick Interferon. J. Bacteriol..

[55] Coppey M., Berezhkovskii A.M., Sealfon S.C., Shvartsman S.Y. (2007). Time and Length Scales of Autocrine Signals in Three Dimensions. Biophys. J..

[56] Swick A., Baltes A., Yin J. (2014). Visualizing infection spread: Dual-color fluorescent reporting of virus-host interactions. Biotechnol. Bioeng..

[57] Ross A., Pompano R. (2018). Diffusion of cytokines in live lymph node tissue using microfluidic integrated optical imaging. Anal. Chim. Acta.

[58] Thurley K., Gerecht D., Friedmann E., Höfer T. (2015). Three-dimensional gradients of cytokine signaling between T cells. PLoS Comput. Biol..

[59] Oyler-Yaniv A., Oyler-Yaniv J., Whitlock B.M., Liu Z., Germain R.N., Huse M., Altan-Bonnet G., Krichevsky O. (2017). A Tunable Diffusion-Consumption Mechanism of Cytokine Propagation Enables Plasticity in Cell-to-Cell Communication in the Immune System. Immunity.

[60] Quinlivan M., Nelly M., Prendergast M., Breathnach C., Horohov D., Arkins S., Chiang Y.W., Chu H.J., Ng T., Cullinane A. (2007). Pro-inflammatory and antiviral cytokine expression in vaccinated and unvaccinated horses exposed to equine influenza virus. Vaccine.

[61] García-Sastre A., Egorov A., Matassov D., Brandt S., Levy D.E., Durbin J.E., Palese P., Muster T. (1998). Influenza A virus lacking the NS1 gene replicates in interferon- deficient systems. Virology.

[62] Kugel D., Pulverer J.E., Köster M., Hauser H., Staeheli P. (2011). Novel Nonviral Bioassays for Mouse Type I and Type III Interferon. J. Interf. Cytokine Res..

[63] Meager A., Gaines Das R., Zoon K., Mire-Sluis A. (2001). Establishment of new and replacement World Health Organization International Biological Standards for human interferon alpha and omega. J. Immunol. Methods.

[64] Ivashkiv L.B., Donlin L.T. (2014). Regulation of type I interferon responses. Nat. Rev. Immunol..

[65] Cardinaud S., Becker C., Kwan W.H., Conrad C., Anguiano E., Albrecht R.A., Iannacone M., García A. (2018). Constitutive resistance to viral infection in human CD141+ dendritic cells. Sci. Immunol..

[66] Sato M., Suemori H., Hata N., Asagiri M., Ogasawara K., Nakao K., Nakaya T., Katsuki M., Noguchi S., Tanaka N. (2000). Distinct and Essential Roles of Transcription Factors IRF-3 and IRF-7 in Response to Viruses for IFN-*α*/*β* Gene Induction. Immunity.

[67] Akpinar F., Inankur B., Yin J. (2016). Spatial-Temporal Patterns of Viral Amplification and Interference Initiated by a Single Infected Cell. J. Virol..

[68] Duvigneau S., Sharma-Chawla N., Boianelli A., Stegemann-Koniszewski S., Nguyen V.K., Bruder D., Hernandez-Vargas E.A. (2016). Hierarchical effects of pro-inflammatory cytokines on the post-influenza susceptibility to pneumococcal coinfection. Sci. Rep..

[69] Mayer-Barber K.D., Yan B. (2017). Clash of the Cytokine Titans: Counter-regulation of interleukin-1 and type I interferon-mediated inflammatory responses. Cell. Mol. Immunol..

[70] Grandvaux N., Servant M.J., TenOever B., Sen G.C., Balachandran S., Barber G.N., Lin R., Hiscott J. (2002). Transcriptional profiling of interferon regulatory factor 3 target genes: Direct involvement in the regulation of interferon-stimulated genes. J. Virol..

[71] Doğanay S., Lee M.Y., Baum A., Peh J., Hwang S.Y., Yoo J.Y., Hergenrother P.J., García-Sastre A., Myong S., Ha T. (2017). Single-cell analysis of early antiviral gene expression reveals a determinant of stochastic IFNB1 expression. Integr. Biol..

[72] Killip M.J., Young D.F., Ross C.S., Chen S., Goodbourn S., Randall R.E. (2011). Failure to activate the IFN-*β* promoter by a paramyxovirus lacking an interferon antagonist. Virology.

[73] Lopez C.B. (2014). Defective Viral Genomes: Critical Danger Signals of Viral Infections. J. Virol..

