# Supplementary material for: Investigating Functional Roles for Positive Feedback and Cellular Heterogeneity in the Type I Interferon Response to Viral Infection
Source: Viruses. 2018 Sep 21;10(10):517. doi: 10.3390/v10100517 (PMC6213501; doi:10.3390/v10100517)
Supplement: Supplementary file 1 [file viruses-10-00517-s001.pdf]

Supplementary Information

S1 Fixed Parameter Values

The tables below show the value of all extended model parameters that were fixed. See the main text for the parameters fitted to data and their confidence intervals.

parameter	value
a	1
δ	0
k	0.015
p	0
c	0
d	0.15
σ	0
V_0	40

Table S1: **Fixed Parameter Values for Rand et al fit**

parameter	value
a	1
δ	0
p	0
c	0
d	0.15
σ	0
V_0	1

Table S2: **Fixed Parameter Values for Patil et al fit**

parameter	value
β	$6.67 * 10^{-6}$
ϕ	0.15
a	1
δ	0.083
k	0.083
c	0.21
d	0.075
V_0	43

Table S3: **Fixed Parameter Values for Saenz et al fit**

parameter	value
a	1
δ	0
k	0.03
c	0.4
d	0.15
s_T	0.041
s_R	0.083
V_0	1500

Table S4: **Fixed Parameter Values for Schmid et al fit** Since Schmid et al studied DENV infection in carcinoma cells, we assumed, following the parameterization in Schmid et al, that the target cell population grew with rate s_T and the restricted cell population grew with rate s_R. The T and R cellular compartment equations in the extended model were accordingly adjusted.

S2 Fits to Data

Model fits are presented in the main manuscript, except for the Schmid et al dataset, which is shown in the two figures below.

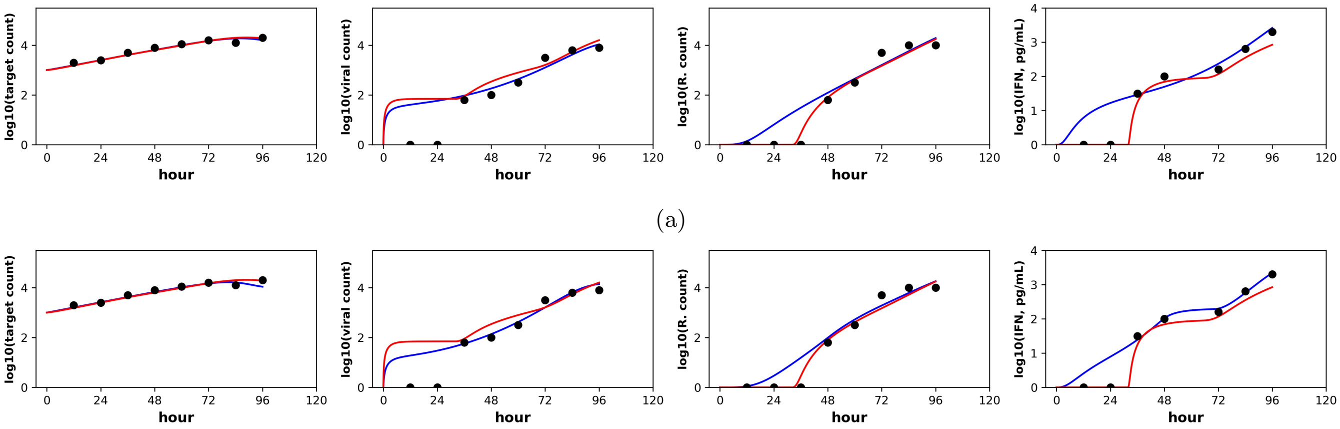

(a)

(b)

Figure S1: **Fit of the Extended Model to Schmid et al WT dataset**. Shown is the fit of our extended model (blue), assuming a (a) constant secretion rate and (b) pulsed secretion rate model, and the fit of the Schmid et al model (red). Panels show, from left to right, the log10 of the total count of cells free of viral protein and IFIT1 expression, the total count of cells with viral protein, and the total count of cells with IFIT1 expression, and extracellur IFN (U), all per ml.

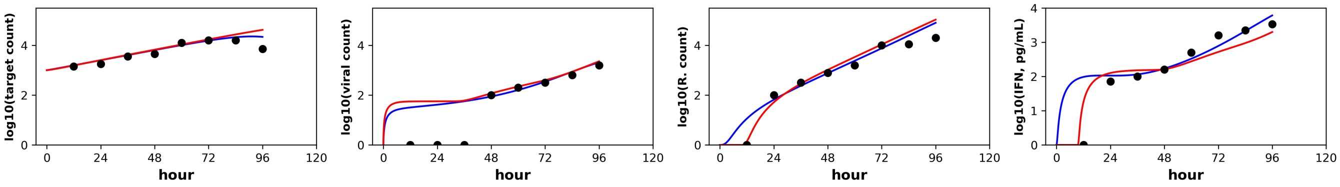

(a)

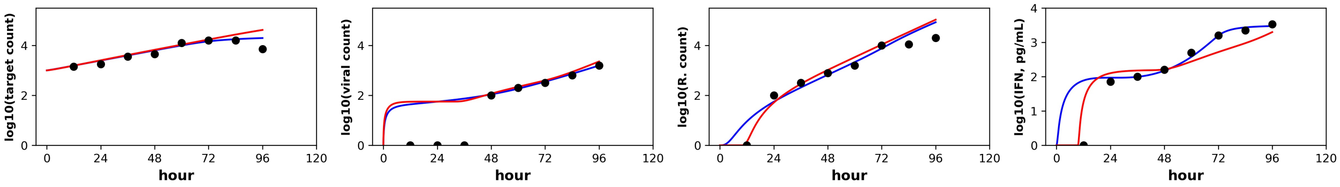

(b)

Figure S2: **Fit of the Extended Model to Schmid et al MT dataset**.

S3 Likelihood Curves

Show below are the likelihood curves used to generate confidence intervals for the Rand et al dataset.

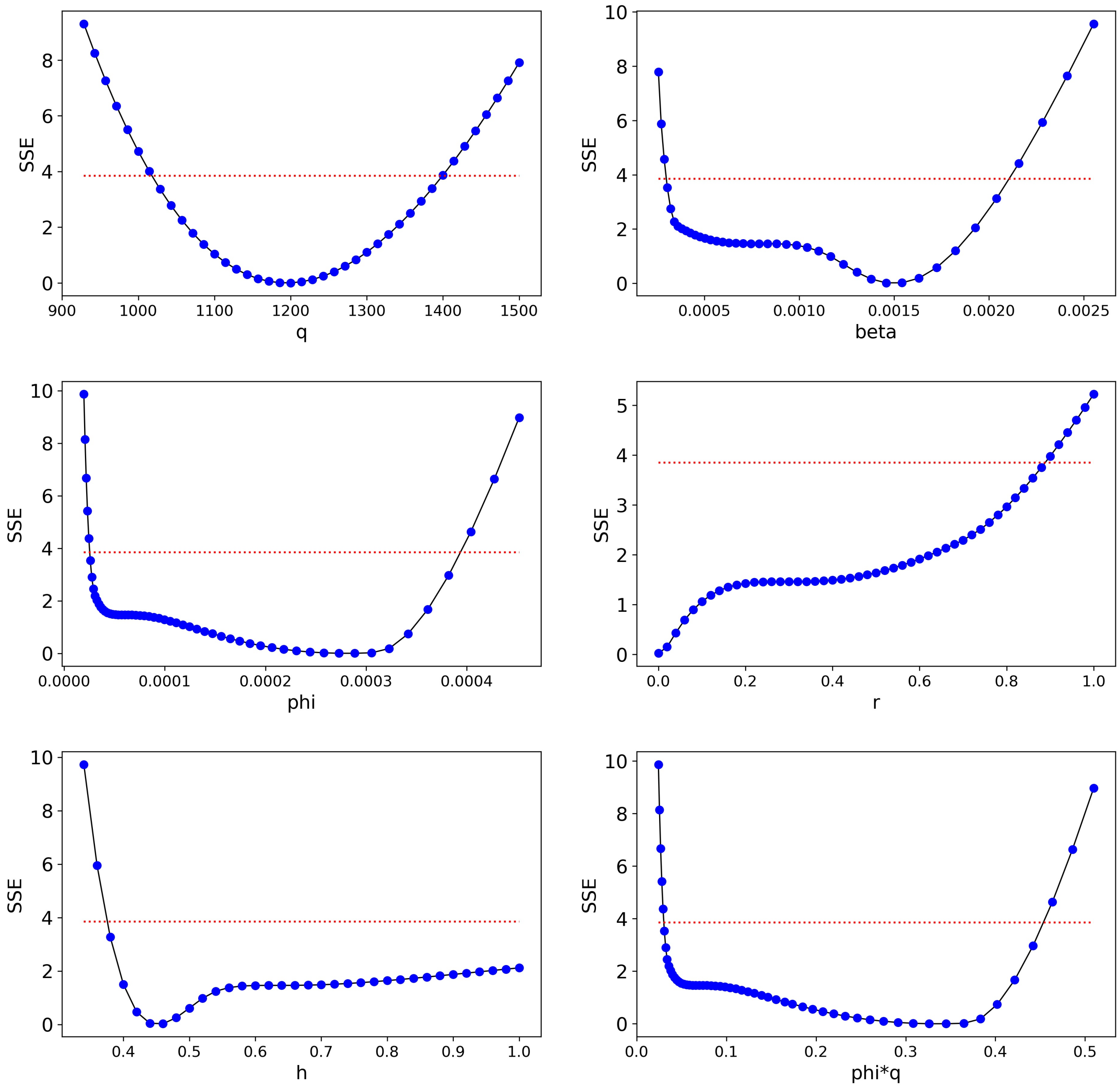

Figure S3: **Likelihood Curves for the Rand et al dataset Under the Constant Secretion Model**. The dashed red line represents statistical significance at 0.05. Each blue dot corresponds to a particular value of the parameter at which we optimized all other parameters and then computed the difference between SSE relative to the SSE of the optimal fit. See Methods in the main text for details.

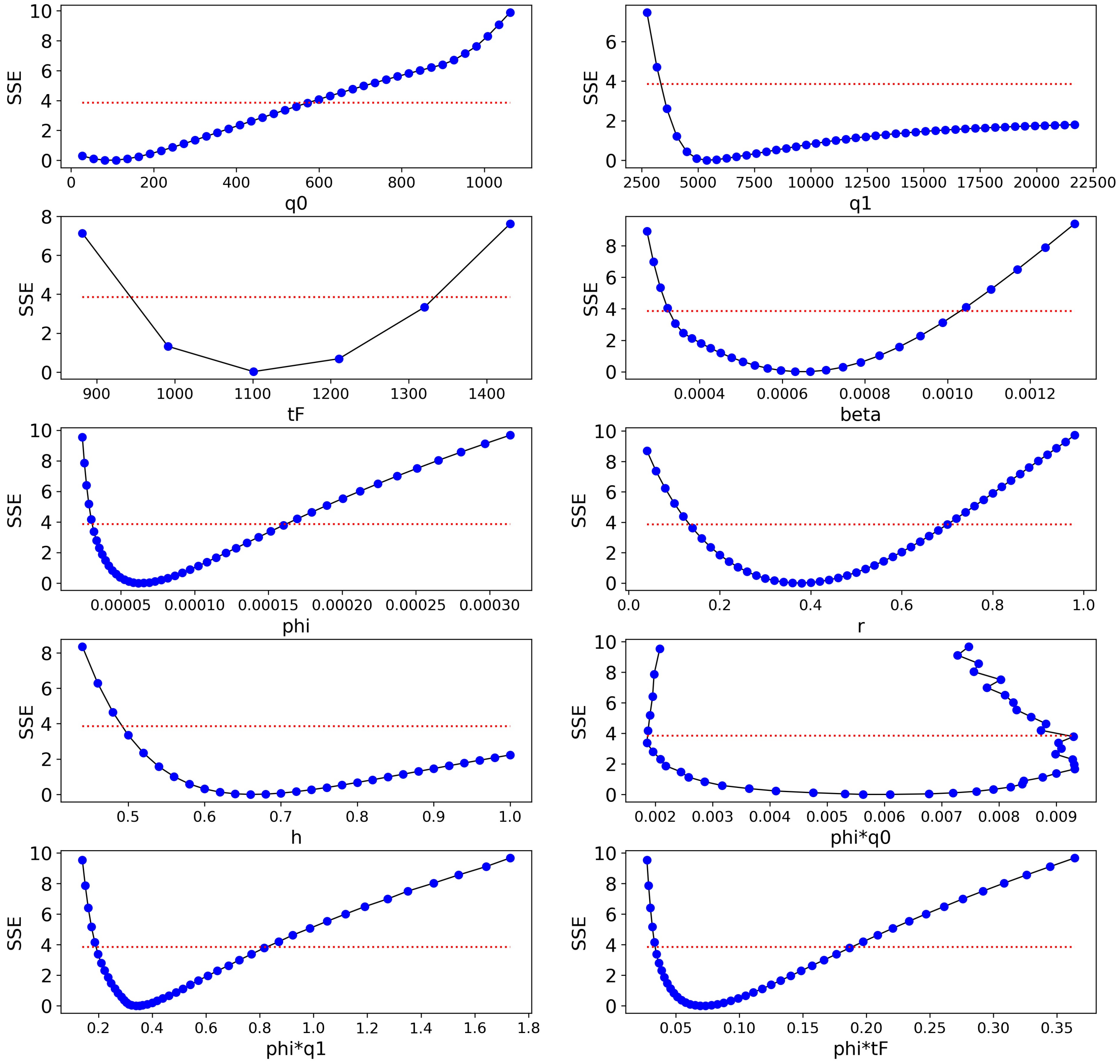

Figure S4: **Likelihood Curves for the Rand et al dataset Under the Pulsed Secretion Model**.
